# Cancer Cells Exploit Notch Signaling to Redefine a Supportive Cytokine Milieu

**DOI:** 10.3389/fimmu.2018.01823

**Published:** 2018-08-14

**Authors:** Michela Colombo, Leonardo Mirandola, Maurizio Chiriva-Internati, Andrea Basile, Massimo Locati, Elena Lesma, Raffaella Chiaramonte, Natalia Platonova

**Affiliations:** ^1^Department of Health Sciences, Università degli Studi di Milano, Milano, Italy; ^2^Kiromic Biopharma Inc., Houston, TX, United States; ^3^Department of Lymphoma and Myeloma, The University of Texas MD Anderson Cancer Center, Houston, TX, United States; ^4^Department of Gastroenterology, Hepatology and Nutrition, The University of Texas MD Anderson Cancer Center, Houston, TX, United States; ^5^Department of Oncology and Hemato-Oncology, Università degli Studi di Milano, Milano, Italy; ^6^Department of Medical Biotechnologies and Translational Medicine, Università degli Studi di Milano, Milano, Italy; ^7^Humanitas Clinical and Research Center, Rozzano, Italy

**Keywords:** Notch, cytokine, chemokine, cancer, immune response, VEGF, inflammation, senescence

## Abstract

Notch signaling is a well-known key player in the communication between adjacent cells during organ development, when it controls several processes involved in cell differentiation. Notch-mediated communication may occur through the interaction of Notch receptors with ligands on adjacent cells or by a paracrine/endocrine fashion, through soluble molecules that can mediate the communication between cells at distant sites. Dysregulation of Notch pathway causes a number of disorders, including cancer. Notch hyperactivation may be caused by mutations of Notch-related genes, dysregulated upstream pathways, or microenvironment signals. Cancer cells may exploit this aberrant signaling to “educate” the surrounding microenvironment cells toward a pro-tumoral behavior. This may occur because of key cytokines secreted by tumor cells or it may involve the microenvironment through the activation of Notch signaling in stromal cells, an event mediated by a direct cell-to-cell contact and resulting in the increased secretion of several pro-tumorigenic cytokines. Up to now, review articles were mainly focused on Notch contribution in a specific tumor context or immune cell populations. Here, we provide a comprehensive overview on the outcomes of Notch-mediated pathological interactions in different tumor settings and on the molecular and cellular mediators involved in this process. We describe how Notch dysregulation in cancer may alter the cytokine network and its outcomes on tumor progression and antitumor immune response.

## Introduction

The critical events in tumor development and progression include heterotypic interactions between neoplastic cells and normal components of the tumor niche. This crosstalk causes the activation of several signaling pathways that, in turn, promote tumor growth, survival, drug resistance, bone resorption, and metastases.

The interplay between tumor cells and immune system has a crucial role in this process. Indeed, tumor development causes a dysregulation of the physiological cytokine milieu, affecting the effectors of cellular and innate immunity, ultimately tipping the balance between immunosuppression and immune stimulation that sustains the disease progression (1).

Recently, Notch signaling has emerged as a key regulator of the cellular relationships within the tumor microenvironment (TME). The Notch system comprises a family of transmembrane receptors (Notch1–4), activated by the interaction with five membrane-bound ligands (Jagged1–2 and Dll1–3–4) present on adjacent cells. Ligand binding results in Notch cleavage by two proteases, ADAM and γ-secretase. These cleavages release Notch intracellular domain (ICN) from the plasma membrane, allowing it to translocate into the nucleus, where it regulates the transcription of a plethora of target genes in a transcriptional complex with the CSL (CBF-1/suppressor of hairless/LAG-1, also known as RBP-Jk), mastermind-like (MAML1–3) coactivator, and other proteins (2). Besides this canonical Notch pathway, in oncogenesis and inflammation, it has been described a non-canonical Notch signaling which is γ-secretase independent (3). Notch signaling is tightly controlled by several mechanisms, including degradation mediated by the proteasome and lysosome machineries (4, 5). The cancer-related aberrant activation of Notch pathway affects the biology of the single tumor cell and its interaction with the surrounding microenvironment (6).

In this review, we analyze how the dysregulation of the Notch pathway in the tumor niche skews the local cytokine milieu (Table 1), shaping the immunological landscape, and we describe the outcomes of this process on tumor growth, progression, senescence, and metastases illustrating the different molecular mechanisms and mediators operating in the distinct cellular contexts.

**Table 1:** Effects of Notch signaling on the cytokine milieu and the immune system.

Notch pathway member	Cytokine	Main functions	Cancer type	Immune mediators	Reference
Notch1	TGFβ	Immunosuppression, anti-inflammatory, epithelial-to-mesenchymal transition, angiogenesis	–	DC, Treg	(7, 8)
Dll4	TGFβ	as above	Lung carcinoma	MDSC	(9)
Notch3, Jagged1	IL-6	as above	Breast cancer	MDSC	(10–12)
Unknown	CXCL12	Migration, proliferation, angiogenesis	Multiple myeloma	M2	(13, 14)
Unknown	CXCL12	as above	Ovarian cancer	T lymphocyte	(15, 16)
Unknown	CXCL12	as above	Hepatocellular carcinoma	Treg, M2	(6, 17)
Dll family, Jagged1/2	IL-10	as above	–	Th1	(18)
Unknown	IL-10	as above	Melanoma, lung carcinoma	TAM	(19–21)
Dll family	IL-10	Immunosuppression, anti-inflammatory	–	DC, Th1	(22, 23)
Jagged1/2, Notch1	IL-4	as above	–	Th2, DC	(24–26)
Dll4	IL-4	Immunosuppression	–	TAM	(27)
Dll4	IL-17	as above	–	γδT cell	(28)
Unknown	IL-17	as above	Oral cancer	CD4+ T, Th17	(29)
Notch1, Jagged2	CCL5	Proliferation, invasion, metastasis	Breast cancer	TAM M2	(30)
Jagged1	IL-1β, CCL2	Pro-inflammatory, proliferation	Breast cancer	TAM	(31, 32)
Notch1	CCL2	Proliferation	Lung carcinoma	Mo-MDSC macrophage	(33)
Jagged1	IFN-γ	Killing immunological functions	–	DC, T cell	(34)
Jagged2	IFN-γ	as above	Lymphoma	NK	(35)
Notch1, Notch2	IFN-γ	as above	–	CD4+ T, CD4+ Th1, CD8+ T	(36–38)
Dll1	VEGF	Angiogenesis, immunosuppression	Lung carcinoma	T cell	(39)

## Notch Signaling Promotes an Immunosuppressive TME

The TME is characterized by the prevalence of anti-inflammatory, immunosuppressive cytokine milieu. The production of an immunosuppressive secretome often requires Notch signaling activation. In this chapter, we explore the role of Notch as a positive regulator of the most important anti-inflammatory cytokines, such as transforming growth factor-β (TGF-β), interleukin 10 (IL-10), interleukin 4 (IL-4), and IL-6. The role of CXCL12 and of receptor activator of nuclear factor kappa-B ligand (RANKL) will be discussed as well.

## Transforming Growth Factor-β

Transforming growth factor-β is expressed at high levels in several malignancies, where it correlates with poor prognosis (40). The main source of TGF-β in cancer is tumor and stromal cells, but it may also be released following bone extracellular matrix remodeling mediated by bone-associated tumors (41).

Transforming growth factor-β supports tumor progression through several mechanisms. The activation of TGF-β receptor promotes chemoresistance and angiogenesis in breast, prostate, gastric, and colon cancer. In addition, TGF-β is also a key player in epithelial-to-mesenchymal transition (EMT) (42).

Transforming growth factor-β is best known for its potent immunosuppressive activity that affects both cells of the innate and adaptive immunity (43–46).

The crosstalk between TGF-β and Notch triggers the TGF-β immunosuppressive activity in several contexts. TGF-β is a well-known inhibitor of DC maturation and, upon stimulation of TGF-βRI receptor, the active form of Notch1 can boost TGF-βRI signaling in DCs by binding Smad3. The interaction of Notch1 with Smad3 promotes the translocation of the latter into the nucleus and induces the transactivation of Smad target genes (7, 42). Moreover, Ostroukhova et al. demonstrated that T-reg cell-derived TGF-β inhibited the activation of effector T cells through the Notch target, HES1. *In vivo* experiments confirmed that this inhibitory effect of Tregs on the activation of effector T cells may be reverted by the treatment with anti-Notch1 antibodies (8).

In lung carcinoma, Notch mediates the pro-tumoral effect of TGF-β secreted by CD11b+ Ly6C+ Ly6G− myeloid-derived suppressor cells (MDSCs). MDSCs are a heterogeneous population of immature myeloid cells that can inhibit T cell responses. In lung carcinoma, MDSCs suppress CD4+ and CD8+ T cell activity (47), secrete TGF-β, which promotes neoplastic cells proliferation and the expression of Dll4. MDSC-derived Dll4 activates Notch in lung carcinoma cells, boosting TGF-β signaling by binding and activating Smad proteins. Consistently, lung cancer cells treated with the Notch inhibitors, DBZ and DAPT, showed a reduced response to TGF-β and a decreased cell growth, indicating that at least in part TGF-β pro-tumorigenic functions are Notch dependent, and suggesting that targeting Notch may represent a promising therapeutic strategy to antagonize TGF-β (9).

Finally, it is worth mentioning that the cooperation between TGF-β and Notch pathway, on top of altering the immune surveillance, promotes EMT (6, 42) in different malignancies, such as ovarian cancer (48) and squamous cell carcinoma (49). Here, high levels of ICN1 seem to cooperate with the TGF-β pathway in the tumor milieu, favoring Smad2/3 phosphorylation, and finally promoting EMT and the survival of tumor-initiating cells (49). The molecular basis of this process is not fully understood, but its implications in cancer progression are clear. EMT process modifies tumor cell behavior, reducing the adhesion to neighboring cells, promoting the invasion through the basement membrane, and finally allowing metastatic dissemination (50).

**Figure 1:**
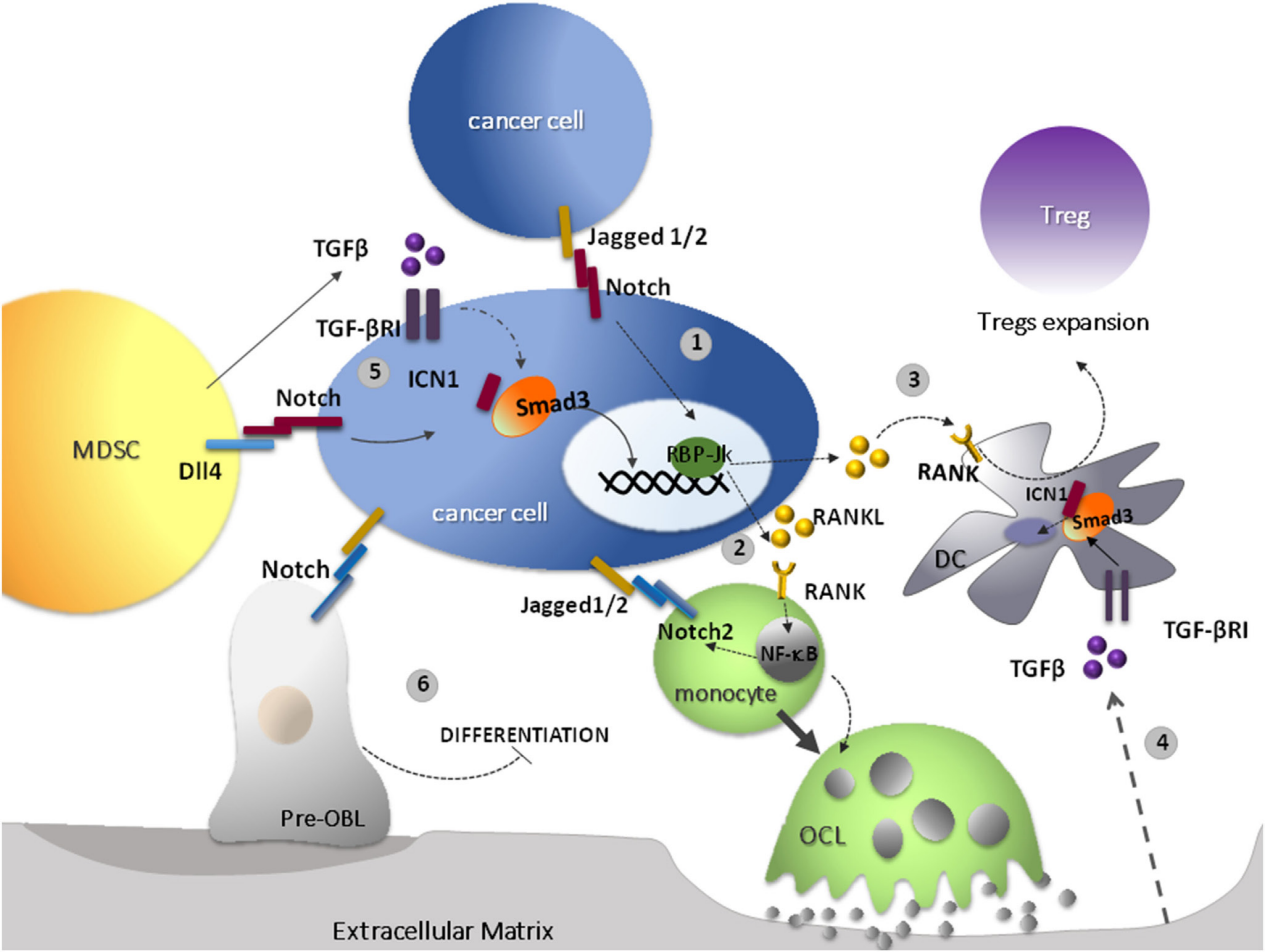
Transforming growth factor-β (TGF-β) and receptor activator of nuclear factor kappa-B ligand (RANKL) cooperate to suppress the immune response in the bone marrow. 1. In bone-associated cancers, the activation of Notch may be promoted by Jagged1/2 ligands overexpressed by cancer cells; one of the outcomes of Notch overactivation is to increase RANKL expression (52). 2. RANKL represents the main osteoclastogenic factor and promotes osteoclasts (OCLs) differentiation and bone resorption (53). 3. In addition, RANKL plays immunoregulatory functions. RANKL may activate its receptor RANK, which is overexpressed by DCs and, in turn, boosts DCs ability to induce the expansion of the local Treg population promoting tolerance to tumor antigens (54). 4. One of the outcomes of the increased bone resorption is the release of TGF-β from the extracellular matrix (55). 5. TGF-β can be also secreted by tumor and stromal cells and by myeloid-derived suppressor cells (MDSCs) in the tumor microenvironment (TME). Its immunosuppressive effects may be promoted by Notch signaling (see text for details) (41, 51). 6. In specific contexts, such as breast cancer-derived bone metastasis, TGF-β released by cancer cells mediates bone remodeling and stimulates the overexpression of Jagged1 in tumor cells. Jagged1 present on cancer cell surface, in turn, triggers Notch activation in OCLs and osteoblasts (OBLs), promoting the development of tumor-associated bone disease (56).

Finally, TGF-β may also positively regulate the Notch pathway through different mechanisms (Figure 1). In breast cancer bone metastasis, Jagged1 acts as a downstream mediator of TGF-β oncogenic signal, contributing to a positive feedback in cancer-mediated bone destruction. Cancer-derived TGF-β mediates bone remodeling and stimulates the overexpression of Jagged1 in tumor cells. In turn, Jagged1, located on the cancer cell surface, triggers Notch activation in osteoclasts (OCLs) and osteoblasts (OBLs). The net effect of this process is OCLs differentiation and activation, and OBLs inhibition (51). This is in agreement with the evidence that Jagged1 forced expression can restore the ability of xenografted breast cancer cells to form bone lesions in Smad knock-out mice (10).

## Interleukin 6

IL-6 has been proposed as a therapeutic target in several tumors, since it represents one of the most abundant soluble factors in the TME (57). It is associated with poor prognosis and is present at high concentrations in the serum of patients with different malignancies, including multiple myeloma, breast, colon, gastric, pancreatic, esophageal, hepatic, cervical, and renal cancer (55, 58).

IL-6 signaling has been shown to promote tumorigenesis by regulating cancer metabolism, increasing cancer cell growth and self-renewal, as well as resistance to apoptosis, boosting invasiveness and metastasis, regulating angiogenesis (57), and sustaining RANKL expression and bone resorption (59). IL-6 may also regulate the immune system by playing a role as pro-inflammatory and anti-inflammatory cytokine (59). Here, we will focus on the immunosuppressive effect, more frequently described in cancer, while we will refer to the pro-inflammatory, immune-activating effect of IL-6 in the chapter on cancer cell senescence.

**Figure 2:**
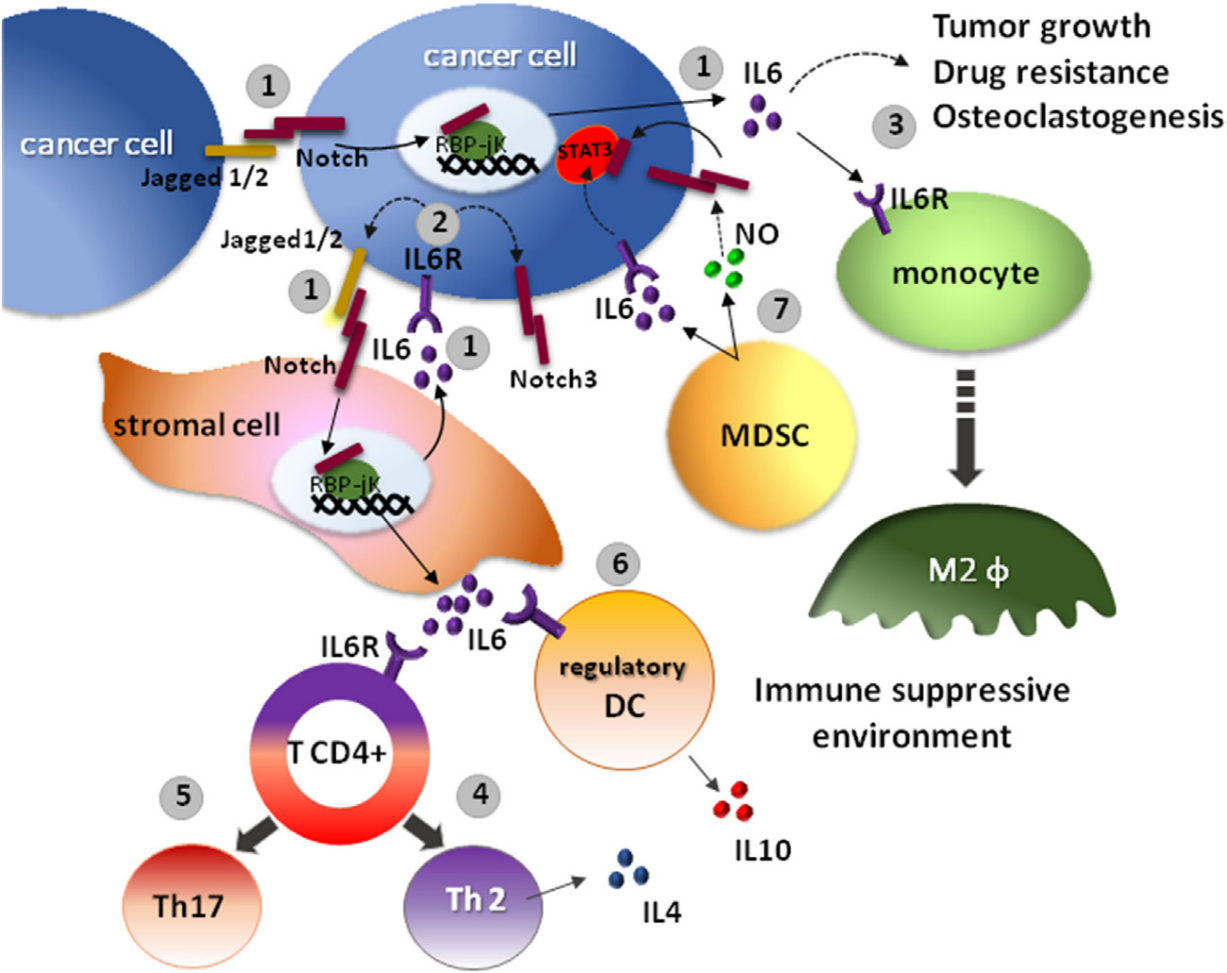
The interplay between IL-6 and Notch affects the anticancer immune response. 1. The overexpression of Notch ligands, Jagged1 and Jagged2 by tumor cells promotes the activation of the Notch signaling in the tumor microenvironment (TME), boosting IL-6 secretion by the same cancer cells and by the neighboring stromal cells (62). 2. On the other hand, IL-6 increases the expression of Jagged1 and Notch3 in tumor cells (64). 3. High IL-6 levels in the TME promote tumor cells growth, resistance to therapy, osteoclastogenesis, and contributes to the development of an immunosuppressive niche. Indeed, the activation of IL-6R on immune cells causes the polarization of M2 macrophages. 4. The differentiation of CD4+ T cells into interleukin 4 (IL-4)-producing Th2 cells. 5. The increased differentiation of pro-inflammatory Th17 cells. 6. The development of interleukin 10 (IL-10)-producing regulatory DCs (65). 7. An alternative source of IL-6 is represented by myeloid-derived suppressor cells (MDSCs). MDSCs support tumor stem cell maintenance by a combined action of IL-6 and nitric oxide (NO). Indeed, MDSCs, through the release of IL-6, promotes the phosphorylation of signal transducer and activator of transcription 3 (STAT3) essential for maintenance of cancer cell stemness, and by producing NO indirectly activates the Notch pathway. Notch activation, in turn, causes prolonged STAT3 activation (12).

The activation of Notch pathway induces the expression of IL-6 in malignant cells of different tumors, i.e., in colon cancer, stimulates tumor cell proliferation (60), and in luminal breast cancer, it promotes self-renewal and drug resistance (61). In other malignancies, such as multiple myeloma (62) and gastric cancer (63), Notch ability to drive IL-6 secretion has been observed also in the surrounding stromal cells of the TME. The increase of IL-6 in the TME promotes tumor cell growth and disease progression (Figure 2).

The interplay between IL-6 and Notch has been studied in depth in multiple myeloma and breast cancer. Myeloma cells colonize the bone marrow (BM), which represents a safe harbor, where tumor cells find an ideal environment for their proliferation and survival (66). In the BM of multiple myeloma patients, IL-6 is produced by tumor cells, BMSCs, and cells of the myeloid lineage, such as eosinophils, macrophages, DCs, and mast cells (67). Recently, we demonstrated that in multiple myeloma the overexpression of Notch ligands, Jagged1 and Jagged2, combined with the expression of Notch receptors, activates the endogenous Notch signaling, which drives IL-6 secretion. Moreover, myeloma cell-derived Jagged may activate Notch receptors in BMSCs *via* heterotypic interaction and promote IL-6 secretion, ultimately causing IL-6 levels to increase in the BM microenvironment (62). In myeloma progression, IL-6 promotes tumor cells growth, osteoclastogenesis, resistance to therapy (62, 67, 68), and, importantly, contributes to the development of an immunosuppressive milieu in the BM niche (67). The mechanism underling the immunosuppressive activity of IL-6 in multiple myeloma is complex and still poorly understood. The outcomes of IL-6 activity on immune cells include favoring the polarization of M2 macrophages, inhibiting Th1 differentiation, and redirecting CD4+ T cells differentiation into IL-4-producing Th2 cells, promoting the differentiation of immature DCs in IL-10-producing regulatory DCs (65). Moreover, IL-6, together with TGF-β, affects the balance between Tregs and Th17 cells, reducing the tumor-suppressive Tregs and promoting the differentiation of pro-inflammatory, Th17 cells (67). Nonetheless, the final outcome of Th17 cells in multiple myeloma is not clear. In different tumor settings, Th17 cells may either positively regulate immune surveillance or promote tumor cells survival (67, 69). Moreover, IL-6 favors the polarization of M2 macrophages. These cells play a crucial role in connecting cancer with inflammation and support tumor cells proliferation, invasion, and metastasis development, promote angiogenesis, and hamper T-cell-mediated antitumor immune response, thus sustaining tumor progression (70).

In breast cancer, high IL-6 is associated with poor prognosis (56). Several biological effects triggered by IL-6 are mediated by Notch signaling activation. Indeed, IL-6 requires Notch3 activity to promote cancer cell invasion and self-renewal (11, 71). This is not the only way by which IL-6 and Notch cooperate in this malignancy. Interestingly, MDSCs are another source of IL-6 in the tumor niche. These cells contribute to tumorigenesis by suppressing T cell activation and promoting stem-like properties of breast cancer cells. These effects are mediated by MDSCs ability to promote the interplay between the Notch signaling and IL-6-dependent signal transducer and activator of transcription 3 (STAT3) activation in cancer cells. MDSCs produce IL-6, which promotes the phosphorylation of STAT3, and the production of nitric oxide, in turn activating Notch signal, which causes prolonged STAT3 activation and supports cancer cell stemness (12).

The crosstalk between Notch pathway and IL-6 in breast cancer cells seems to be mediated also by NF-κB. Indeed, the activation of the non-canonical Notch signaling mediated by two components of the NF-κB cascade, IKKα and IKKβ, has been reported to upregulate IL-6 expression (72). The relevance of the non-canonical-Notch/NF-κB/IL-6 axis stems from the evidence that, while canonical Notch4 is necessary for the development of mammary glands, non-canonical Notch4 signaling is related to breast cancer tumorigenesis (73).

The interplay between Notch and IL-6 is even more complicated in breast cancer-associated bone metastasis. Here, the overexpression of Jagged1 activates Notch signaling in BMSCs, promoting the secretion of IL-6. In turn, IL-6 increases the expression of Jagged1 and Notch3 in tumor cells (64) and stimulates tumor growth and drug resistance (10).

Although the above reported findings do not provide a direct evidence, they allow us to hypothesize that the remodeling of the immune system may represent one of the mechanisms through which IL-6 and the Notch pathway cooperate to promote multiple myeloma and breast cancer progression.

## CXCL12

CXCL12, known also as stromal-derived factor 1 (SDF1), binds two chemokine receptors: CXCR4 and CXCR7. We will focus on CXCR4 since it represents the most widely expressed chemokine receptor in human malignancies and it is a crucial player in the plasticity and alteration of the TME both in hematologic tumors, such as multiple myeloma, acute myeloid leukemia, T cell acute lymphoblastic leukemia (T-ALL), and in solid tumors such as ovarian, prostate, colon, brain, breast, and bladder cancer (74–76).

CXCR4 signaling is upregulated by hypoxia or in response to steroid hormones and it is associated with an invasive and metastatic phenotype (77) due to its involvement in several aspects of tumor development and progression such as cell migration, proliferation, resistance to apoptosis, angiogenesis, and development of metastasis (77–80).

In addition, the CXCR4/CXCL12 axis plays also a key role in inducing TME tolerogenic polarization in different types of cancers, although the exact mechanism has not been elucidated. Feig et al. demonstrated that in pancreatic cancer the blockade of CXCL12 produced by tumor-associated fibroblasts promotes CD3+ T-cells recruitment and restores the sensitivity to the antagonists of the checkpoint inhibitors programmed cell death-1 (PD-1) and cytotoxic T-lymphocyte-associated protein 4 (CTLA-4) (81). Accordingly, Chen et al. showed that CXCR4/CXCL12 blockade synergized with anti-PD-L1 immunotherapy in advanced hepatocellular carcinoma (82), while a similar mechanism was recently reported in an *in vivo* model of colorectal cancer (83). This synergy is relevant since, although checkpoint inhibitors have emerged as effective new therapeutic approaches in cancer, the response rate in patients is still variable and could benefit from a combinatory therapy (84).

The cooperation between Notch and CXCR4/CXCL12 has been reported in hematologic and solid malignancies. We recently showed that the expression of CXCR4 and CXCL12 in multiple myeloma cells is positively regulated by Notch signaling and may be impaired by γ-secretase inhibitors (13). The activation of Notch signaling in multiple myeloma is due to the contemporary expression of Notch ligands and receptors (13). Distinct reports indicate that activated Notch promotes CXCR4 gene expression by binding to CXCR4 regulative regions and transactivating its transcription (13, 85). CXCR4/CXCL12 blockade results in a decreased tumor cell proliferation and survival and, importantly, in the loss of myeloma cells ability to colonize the BM *in vivo* (13).

The interaction between Notch, CXCL12, and CXCR4 might also have a further outcome since high CXCL12 levels in the multiple myeloma niche increase the M2 macrophage population in the immune cell infiltrate. Indeed, CXCR4 directs the recruitment of monocyte precursors at the tumor site, and M2 macrophages from the BM of myeloma patients express higher levels of CXCR4 compared with patients with the benign form of monoclonal gammopathy of uncertain significance and healthy individuals (14). Recently, Fabbri et al. demonstrated that also in B-cell chronic lymphocytic leukemia Notch1 is able to directly regulate CXCR4 expression (86), while in other hematological malignancies characterized by Notch1 hyperactivation, such as T-ALL, no Notch1-dependent regulation has been observed, but a cooperation the two pathways. Indeed, CXCR4 genetic deletion in murine hematopoietic progenitors abrogated ICN1 ability to induce leukemogenesis (87), but DAPT treatment failed to inhibit CXCR4 expression either in cell lines or primary cells (88), suggesting that an indirect and more complex mechanism of cooperation between these two pathways may be crucial in promoting tumor progression.

Among solid tumors, ovarian cancer shows a cooperation between Notch and CXCR4 signaling. Indeed, DAPT-mediated Notch inhibition causes a decrease in tumor cells growth and migration through the downregulation of CXCR4 and CXCL12 expression (15). By regulating this chemokine system, Notch might influence also the immunosuppressive function exerted by CXCR4 signaling in ovarian cancer. Indeed, CXCL12/CXCR4 blockade reduces infiltrated Tregs, increases the presence of IFN-γ+/IL-10+ T CD4+ and CD8+ lymphocytes, and supports spontaneous humoral and cellular antitumor responses (16). Similarly, in hepatocellular carcinoma, characterized by persistent Notch activation (6), hypoxia may induce CXCL12 upregulation, that in turn promotes the recruitment of Tregs and M2-type macrophages (17). This suggests that the collaboration between CXCR4/CXCL12 and Notch might induce an immunosuppressive TME involving various types of immune cells among which Tregs and M2 macrophages.

## Receptor Activator of Nuclear Factor Kappa-B Ligand

*RANKL* is a member of the tumor necrosis factor (TNF) family of cytokines. Its deregulation is particularly relevant in bone-associated cancers (primary or secondary) due to its involvement in the maturation of monocyte in OCLs (53) and the resulting associated osteolysis. The increase in RANKL levels characterizes almost all bone-associated cancers such as multiple myeloma and metastases derived from primary tumors which spread to the skeleton, i.e., carcinomas of the prostate, breast, lungs, thyroid, bladder, and kidneys as well as melanoma (89).

Indeed, one of the outcomes of cancer cells localization in the BM is the unbalance between bone destruction and formation due to altered differentiation and activity of OBLs and OCLs (90). This dysregulation is caused by an increased secretion of RANKL by neighboring stromal cells and infiltrating Th17, Tregs and DCs (91), and leads to the development of osteolytic lesions that, not only affect patient’s quality of life, but also promote tumor growth, survival, metastasis formation, and the development of pharmacologic resistance (89, 90, 92, 93) (Figure 1).

Notch pathway dysregulation is involved in several bone-associated tumors. Results from our group and the group of Kang (10, 52) showed a similar situation in multiple myeloma and breast cancer. In both cases, tumor cells overexpress the Jagged ligands and are able to activate the Notch2 receptor in OCL precursors promoting their differentiation, that finally results in increased bone resorption and in the development of bone disease (10, 52). We also showed that in myeloma cells Notch activity positively influences the release of *RANKL*, while Notch inhibition, mediated by gamma secretase inhibitor (GSI) or Jagged ligands knockdown, downregulates RANKL secretion with consequently decreased OCL differentiation and activity (52).

Myeloma-derived RANKL promotes osteoclastogenesis by activating in OCL precursors two major pathways essential for their differentiation. NF-κB is triggered by RANK and Notch signaling is promoted as a consequence of an increase in the expression of Notch2, that in turn is activated by Jagged ligands expressed on myeloma cells (52, 94).

Another Notch-dependent source of RANKL in the BM niche is represented by osteocytes. In these cells, Notch signaling can be activated by the interaction with myeloma cells. As a consequence, Notch activity hampers osteocytes viability and promotes RANKL and sclerostin secretion that, in turn, supports the recruitment of OCL precursors (95).

Although Notch may act as a regulator of the balance between OCL and OBL/osteocyte activity, up to now it has not been investigated if Notch controls also other RANKL activities. Indeed, RANKL is involved in the shaping of the immune system operated by cancer cells in different tumors. RANKL favors the expansion of the local Treg population in bone metastatic prostate cancer (96), promotes M2–macrophages polarization in breast cancer models of lung metastasis (97, 98), interferes with NK anticancer activity in acute myeloid leukemia (51), is necessary for T cell tolerance in a melanoma model (99), where successful results were obtained by a combinatory treatment of RANK/RANKL blockade with anti-CTLA-4 (100). The positive results of preclinical studies induced to design clinical trials to evaluate the potential combinatorial effect of anti-RANKL monoclonal antibodies and immune checkpoint inhibitors (101). Interestingly, all tumors in which RANKL exerts an immunosuppressive activity share a recognized oncogenic activity of Notch, suggesting a collaboration of Notch and RANK also in hampering the antitumor-immune response.

## Interleukin 10

Interleukin 10 is an immunosuppressive anti-inflammatory cytokine produced by various types of cells during the immune response. IL-10 signaling requires the assembly of a two-receptor complex consisting of two copies each of IL-10R1 and IL-10R2 chains; IL-10 receptor binding activates the JAK signal transducer and STAT pathway (102).

Interleukin 10 is produced by Th2 cells and monocytes, as well as by subsets of T cells, namely CD4+ CD25+ Foxp3+ (Tregs) and CD4+ CD25− Foxp3− type 1 regulatory (Tr1), Th1 and CD8+ T cells, B cells, macrophages, DCs, eosinophils, and mast cells (103). Tumor cells produce large amounts of IL-10 that contributes to tumor progression; in most types of cancers, serum IL-10 levels correlate with disease severity (104, 105). In TME, IL-10 secreted by immune and malignant cells activates an autocrine loop that relies on IL-10 receptor and induces the upregulation of oncogenes, including cancerous inhibitor of protein phosphatase 2A and MYC (106).

Interleukin 10 released in TME exerts its immunosuppressive function in different ways: (1) unbalancing Th1 vs Th2 tumor-specific immune responses; (2) mediating the differentiation and activation of Tr1 cells involved in immunosuppression (107, 108); (3) inhibiting the production of pro-inflammatory cytokines and mediators such as IL-1, IL-6, IL-12, and tumor necrosis factor α (TNFα) by macrophages and DCs; (4) preventing the differentiation of DCs from monocytes and their maturation (109). In particular, IL-10 downregulates MHC-II on DCs and the co-stimulatory molecules CD80 and CD86 on macrophages (110); therefore, DCs display a defective antigen presentation and fail to activate cytotoxic T cells (111). Collectively, these effects promote the progression in different tumor such as ovarian carcinoma, lymphoma, and melanoma (102, 112).

Recently, an antithetic immunostimulatory function of IL-10 has been reported, too. Indeed, IL-10 may also promote the proliferation of CD8+ T cells (113), the differentiation of plasma cells along with the prolongation of their survival, the proliferation of NK cells, and their production of IFN-γ upon stimulation with IL-18 (114, 115). It is possible that the overall effect of IL-10 depends on the specific tumor type and TME, therefore a targeted therapy directed to IL-10 should carefully consider the possible dual immunosuppressive and immunostimulatory role of this cytokine.

The Notch–IL-10 axis is generally involved in self-limitation of immune response. Rutz et al. showed that Notch signaling, in synergy with IL-12 or IL-27, stimulates Th1 cells to release large amounts of IL-10, that contribute to self-limitation of Th1 immunity by hampering the inflammatory potential of Th1 cells (22). Interestingly, only Dll, but not Jagged ligands expressed by DCs are able to trigger Notch receptors located on T cells surface and activate IL-10 production *in vitro* and *in vivo* (22, 23).

Recently, a negative feedback regulation of hepatic inflammation mediated by the Notch–IL-10 axis was also reported. Hepatic inflammation is associated with the expression of Dll and Jagged ligands in liver sinusoidal endothelial cells that, in turn, activates Notch signaling in Th1 cells, with a consequent increase in HES1 and Deltex-1 expression (18). Notch activation triggers the production of IL-10 in Th1 cells causing their switch from an inflammatory to an immunosuppressive function. Consistently, Notch-deficient CD4+ T cells express lower IL-10 levels in the presence of liver sinusoidal endothelial cells, leaving the expression of Th1 cytokines, such as IFN-γ and TNFα, unaltered (18). We speculate that this mechanism of self-limitation of T-cell response in inflamed liver might also occur in hepatocellular carcinoma since it arises in more than 90% of cases as consequence of hepatic injury and inflammation (116).

So far, we have reported an inhibitory role of the Notch–IL-10 axis on the immune system mediated by Th1 cells and T cells activation, but unexpectedly, this axis also acts to switch tumor-associated macrophages (TAMs) to the inflammatory, antitumor phenotype M1, thereby increasing the antitumor immune response. Indeed, the conditional expression of ICN in macrophages of a transgenic murine model induces the conversion of TAM from M2 to M1 phenotype by inducing the expression of miR-125a, resulting in TNFα and IL-12 secretion and reduced release of IL-10 and TGF-β. These macrophages exhibited strong antitumor activities in transplanted tumors (19). Conversely, Notch blockade by GSI, small interfering RNA, or RBP-Jk deletion switches macrophages to M2 phenotype, characterized by the ability to produce IL-10 and an attenuated capacity of activating Th1 cells (20, 21). Consistently, T cells activated by RBP-Jk−/− macrophage showed a reduced cytotoxic activity against melanoma cells when compared with wild-type macrophages (20).

## Interleukin 4

In the TME, IL-4 is produced by tumor cells, mast cells, activated Th2 cells, eosinophils, basophils, and MDSCs (117, 118). A close relationship between tumor progression and IL-4 produced by tumor-infiltrating Th2 lymphocytes has been found in several malignancies such as non-small cell lung carcinoma, breast cancer, renal cell carcinoma, prostate cancer, and others (117). Moreover, enhanced expression of the IL-4 receptor (IL-4R) has been reported in various neoplastic tissues, i.e., glioblastoma, malignant melanoma, head and neck cancer, renal cell carcinoma, breast, prostate, ovarian cancer, and bladder cancer (117, 119).

Interleukin 4 signaling supports cancer cell proliferation and survival (120). Moreover, IL-4 contributes to suppress the antitumor immune response by acting at different levels on adaptive and acquired immune system (121).

Although no evidence has been reported of an interaction between Notch and IL-4 in cancer cells, Notch signaling plays a key role in activating IL-4 expression in different cellular components of the TME. A Notch/RBP-Jk binding site has been identified in the 3′ end of the IL-4 gene, suggesting that the Notch pathway has the ability to directly regulate IL-4 expression (122); moreover, Notch has been shown to control IL-4 secretion in myeloid progenitors and NK cells probably due to the presence of two RBP-Jk binding sites in the conserved non-coding sequence-2, located downstream the IL-4 gene (123). The cooperation between Notch and IL-4/IL-4R pathway also contributes to the differentiation and activation of immunosuppressive Th2 cells. The activation of Notch signaling by DC-expressed Jagged2 induces Th2 cells differentiation by boosting the expression of GATA3, IL-2/IL-2Rα, and IL-4 (24, 124). A previous work from Fang et al. suggest that Notch1 is the receptor involved in this process, since ICN1 directly regulates GATA3, which is a master regulator of Th2 differentiation and promotes IL-4 transcription by coordinating chromatin remodeling (25, 125). Sauma et al. demonstrated that IL-4 produced by Th2 cells may also promote a positive feedback loop on T cell polarization sustaining Jagged2 expression in DCs (24).

The existence of a crosstalk between Notch and IL-4 signaling during DCs differentiation is supported by the findings of Cheng et al., who showed that Jagged1-induced Notch activation causes the accumulation of immature DCs, because of the lack of IL-4, which is required for their differentiation (26). Conversely, Dll4-mediated Notch activation in macrophages may have an antitumor effect as shown by the evidence that Dll4-mediated Notch1 activation hampers IL-4-induced M2 polarization and promotes M1 macrophage apoptosis; the authors suggest that the interaction between Notch and IL-4 pathway may involve HES1 ability to bind STAT3, finally inhibiting IL-4R signaling (27).

## Notch Signaling Stimulates Cancer-Associated Pro-Inflammatory Cytokines

Cancer is tightly associated with chronic inflammation. The ability of infiltrated immune cells to promote tumor growth, progression, and immune surveillance may be mediated by several pro-inflammatory cytokines and chemokines, including TNFα, interleukin 17 (IL-17), IL-1, and CCL2 and CCL5. In the following sections, we will describe the interaction of Notch signaling with such cytokines and the outcome of their interplay on the immune response.

## Tumor Necrosis Factor α

Tumor necrosis factor α is a pro-inflammatory and immunomodulatory cytokine, member of the TNF/TNF receptor superfamily (126). TNFα is one of the most strong activators of NF-kB pathway (127). TNFα may be produced in response to inflammation and infection by macrophages, lymphocytes, fibroblasts, and keratinocytes, but also tumor cells may be a relevant source (128).

The pro-inflammatory and immunomodulatory effect of TNFα is at the basis of its pro-tumor activity, observed in different malignancies including cutaneous, ovarian, pancreatic cancer, and tumors of the pleural cavity and the bowel (128). High levels of TNFα in the serum have a poor prognosis in ovarian, renal, pancreatic, prostate, breast cancer, and chronic lymphocytic leukemia (126). The mechanism underlying TNFα pro-tumor activity was nicely described in ovarian cancer. TNFα released by tumor cells and cells of TME acts through its receptor TNFR1 and further reinforces TNFα expression and the inflammatory and immune-modulatory network including CXCL12, CCL2, IL-6, VEGF, and macrophage inhibitory factor (129). This induces the differentiation of myeloid progenitors to endothelial cells, extracellular matrix remodeling, and recruiting leukocytes at tumor site for local immunosuppression (128). TNFα immunosuppressive function includes downregulation of TCR signaling and DC function, promotion of T cell apoptosis, activation of Tregs, induction of tumor cell dedifferentiation with a consequent reduced expression of immunogenic antigens and impaired recognition by cytotoxic T cells, impaired differentiation of immature MDSCs with increased suppressive activity resulting in T and NK cell dysfunction and finally inducing other cytokines that can inhibit cell-mediated immunity (130–134).

**Figure 3:**
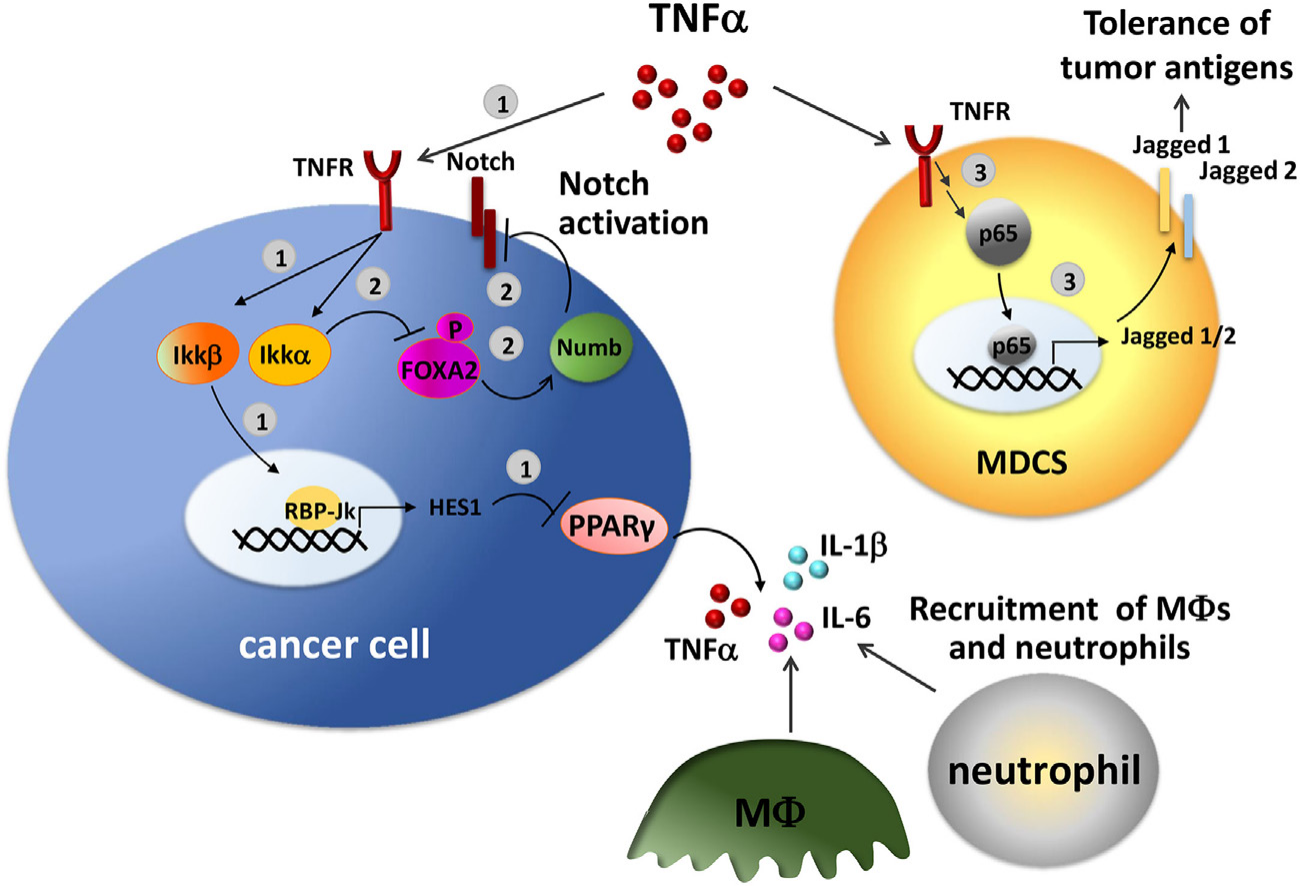
Crosstalk between Notch and tumor necrosis factor α (TNF-α) in the tumor environment. TNF-α induces Notch activation through NF-κB pathway in tumor cells. 1. TNF-α activates Ikkβ inducing the expression of the Notch target gene HES1. HES1, in turn, inhibits the expression of the anti-inflammatory receptor peroxisome proliferator-activated receptor γ (PPARγ) with a consequent increased production of TNF-α, IL-6, and IL-1β, that recruit macrophages and neutrophils in the tumor site (135). 2. TNF-α promotes Notch signaling by activating Ikkα, that in turn phosphorylates and inhibits FOXA2, thereby reducing the transcription of the target gene Numb, a Notch repressor (136). 3. In tumor-infiltrating myeloid-derived suppressor cells (MDSCs), NF-κB-p65, a key mediator of TNF-α/TNFR signaling, transactivates Jagged1 and 2 promoters and induces their expression stimulating the tolerogenic activity of tumor-associated MDSCs (137).

An interplay of Notch pathway and TNFα has been described in several studies (Figure 3). TNFα stimulation may result in the transcription of important Notch target genes mediated by the activity of NF-kB. In a mouse model of pancreatic cancer, TNFα induces the activation of Ikkβ, a component of the NF-κB signaling, that promotes the expression of Notch target genes HES1 by inducing histone H3 phosphorylation at the HES1 promoter resulting in transcriptional activation. This, in turn, inhibits the expression of the anti-inflammatory nuclear peroxisome proliferator-activated receptor γ (PPARγ), reinforcing the inflammatory loop (135). PPARγ repression in pancreatic cancer cells results in the constitutive production of pro-inflammatory cytokines, including TNFα, IL-6, and IL-1β, relevant in the recruitment of macrophages and neutrophils into the tumor site. Consistently, *in vivo* treatment with a PPARγ agonist, rosiglitazone reduces macrophage infiltration (135).

Evidence obtained in liver cancer clearly shows a cooperation between TNFα and Notch in inflammation-mediated cancer pathogenesis. TNFα regulation of Notch1 signaling is mediated by Ikkα-induced phosphorylation of FOXA2. This causes the inhibition of FOXA2 activity as a transcription factor and consequently decreases the expression of its target genes including Numb. Numb is a well-known Notch repressor, thereby the consequence of its inhibition is the increased activation of Notch1, that is associated with tumorigenesis (136). Indeed, by *in vivo* studies on mice that received transplanted tumors harboring Numb knockdown Hep3B cells infected with retrovirus expressing FOXA2 continued to show tumor growth even in the presence of FOXA2. Moreover, a link of IKKα-mediated FOXA2 phosphorylation to hepatocellular carcinoma tumorigenesis was supported by higher levels of IKKα, phosphorylated FOXA2, and activated Notch1 in hepatocellular carcinoma specimens respect to normal liver tissues (136).

Further studies performed on murine models of Lewis lung carcinoma, colon carcinoma, thymoma, and melanoma, showed that the expression of NF-kB-p65, a key mediator of TNFα, is associated to increased levels of Jagged1 and 2 in tumor-infiltrating MDSCs (137), suggesting that TNFα may positively regulate the expression of Jagged ligands. Jagged1 and Jagged2 widely affect immune system regulation as shown *in vivo* with humanized anti-Jagged1/2-blocking antibody CTX014. This treatment affected the accumulation and tolerogenic activity of MDSCs in tumors and inhibited the expression of immunosuppressive factors arginase I and inducible nitric oxide synthase (137). As a consequence, tumor-induced T-cell tolerance was reduced and the infiltration of reactive CD8+ T cells was increased thus enhancing the *in vivo* efficacy of T-cell-based treatment (137).

Finally, one elegant study showed a complementary effect of Notch and TNFα in multiple myeloma-induced bone disease (95). The authors showed that co-cultured multiple myeloma cells and osteocytes reciprocally activated Notch signaling. Notch activation in osteocytes induced apoptosis that in a second phase was amplified by high levels of TNFα secreted by MM cells. The cooperation of the two pathways is further confirmed by the evidence that single treatment with GSI-XX or anti-TNFα only partially inhibited cell death, whereas the combined treatment completely prevented osteocyte apoptosis. The increased apoptosis levels in osteocytes not only reduce their bone deposition activity but also increase active bone matrix degradation since it is also associated with the enhanced expression of the key osteoclastogenic factor RANKL (95). This evidence suggests that multiple myeloma cells exploit the collaboration of Notch and TNFα signaling pathways to induce bone resorption in multiple myeloma and possibly RANKL-mediated immunosuppression.

## Interleukin 17

Interleukin 17 is a family of pro-inflammatory cytokine (including IL-17A to F) mainly produced by Th17 cells, a lineage of T helper cells defined by their ability to produce IL-17, IL-21, and IL-22 (138, 139). Other immune cells may contribute to IL-17 levels, including CD8+ T cells, NK cells, γδ T cells, and neutrophils (140). IL-17 engages one of its five cell surface receptors (IL-17 receptor A to E) and triggers the production of various pro-inflammatory cytokines and chemokines that recruit monocytes and neutrophils to the site of inflammation (141).

The most important role of IL-17 is attributed to its ability to stimulate various cell types to produce pro-inflammatory cytokines and chemokines by activating the NF-κB pathway (142–144).

The presence of Th17 cells and the expression of IL-17 have been found in almost all tumors (145, 146). Nonetheless, the role of IL-17 in cancer is controversial, since both pro-tumoral and antitumoral effects have been reported, possibly due to its pleiotropic activity. IL-17 may promote tumorigenesis in several types of cancer and in different ways: (1) by inhibiting tumor apoptosis and promoting tumor proliferation (147–149); (2) by inducing tumor–associated stroma to release of the pro-tumoral cytokine IL-6 (150); (3) by recruiting macrophages and MDSCs to the tumor site (151, 152); (4) by promoting Tregs infiltration into tumor tissue through upregulation of CCL17 and CCL22 (153); (5) by promoting angiogenesis through the increase of VEGF production (154); and (6) by stimulating tumor cells to express matrix metalloproteinase-2 (MMP-2) and MMP-9 involved in cancer cell invasion (155).

Despite these pieces of evidence, recent studies on cancer patients highlight an antitumoral role of IL-17. Indeed, the 5-year survival rate in patients with gastric adenocarcinoma (156), esophageal squamous cell carcinoma (157), chronic lymphocytic leukemia (158), ovarian cancer (159), and cervical adenocarcinoma (160) displaying increased IL-17 levels was reported to be significantly higher than survival in patients with lower IL-17 expression. The possible mechanisms underlying this effect could rely on the positive regulation of the adaptive immune response *via* stimulation of the production of cytokines and chemokines such as IFN-γ, CXCL9, and CXCL, recruitment of CD4+, CD8+ T cells (159), DCs (157), and neutrophils (161) to tumor sites, stimulation of NK cell activity (157), generation and activation of CTLs (162).

The complex array of IL-17 effects on the immune system response might explain its dual behavior and, probably, its overall outcome may depend on tumor cell type and the surrounding TME, including the pattern of immune cells and cytokines. On the other side, it cannot be ruled out that opposite effects of IL-17 reported for the same type of tumor can be due to differences in the animal models used or the number of cases, or to possible different targets of the performed investigation, including IL-17 produced only by Th17 cells or by the whole the set of IL-17-producing subsets of cells.

**Figure 4:**
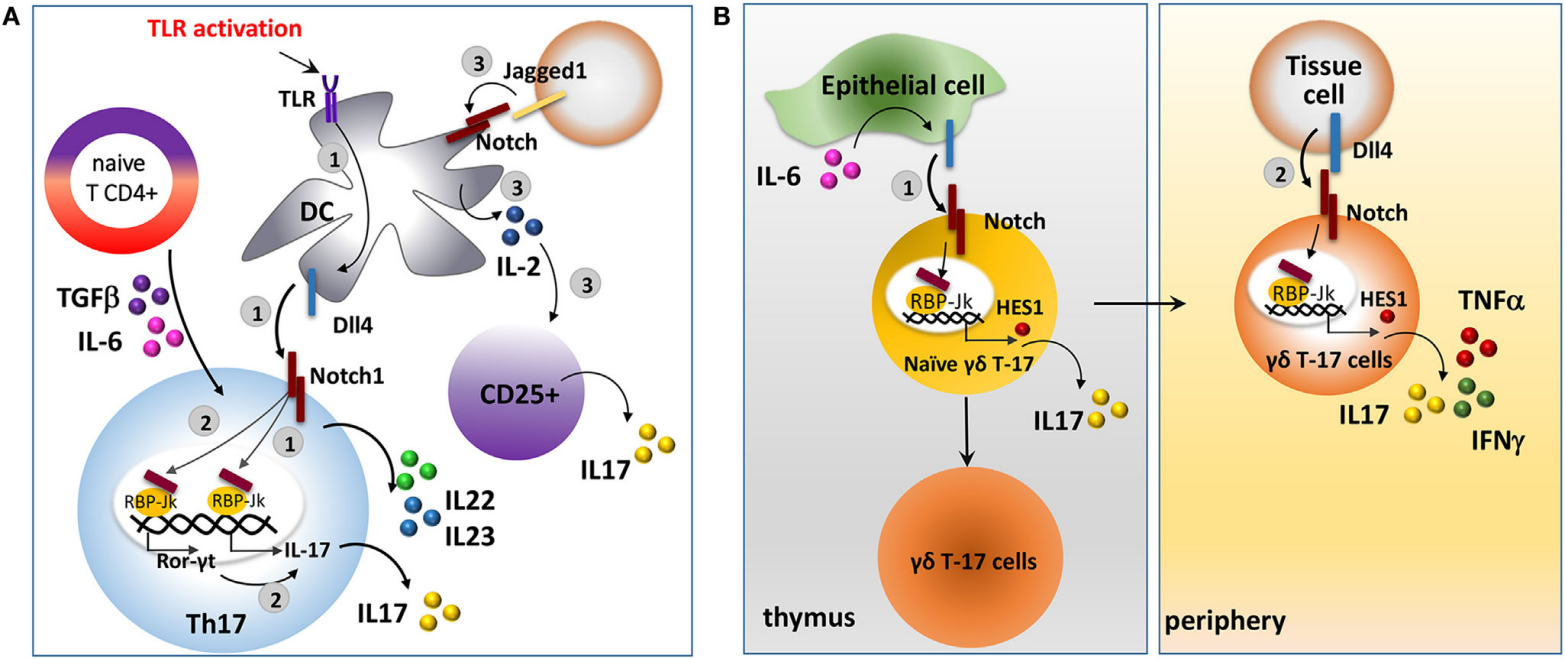
The interaction between Notch and interleukin 17 (IL-17) in different immune cellular settings. **(A)** Notch signaling stimulates Th17 cell differentiation. 1. Dll4, upregulated on DC cells upon TLR activation, in cooperation with skewing cytokines, IL-6 and transforming growth factor-β (TGF-β), activates Notch signaling in naive CD4+ T cells inducing the production of IL-17 and Th17-related cytokines, IL-22 and IL-23, and the consequent differentiation in Th17 cells. Dll4-mediated Notch activation in naive CD4+ T cells results in the direct transactivation of IL-17 promoter by activating the RBP-Jk/CSL transcription factor (163–165). 2. In addition, ICN may also stimulate Th17 cell differentiation by transactivating the promoter of Ror-γt, a transcription factor required for Th17 differentiation and a main regulator of IL-17. 3. Jagged1-conditioned DCs produce IL-2 that is required for the production of IL-17 by CD25+ cells (166). **(B)** Notch signaling in γδ T-17 cells. 1. Dll4, upregulated on thymic epithelial cells following IL-6 induction, activates Notch signaling in γδ T-17 cells resulting in expression of HES1, that in turn induces the production of IL-17 and the development of γδ T-17 cells (28, 167). 2. High level of Dll4 in peripheral tissues, such as the intestine and lung, is enable to activate Notch in γδ T-17 cells (28). This results in the production of IL-17, IFN-γ, and tumor necrosis factor α (TNFα) along with the proliferation and activation of peripheral γδ T-17 cells and enhancement of their antitumor cytolytic effect (29).

Notch regulates IL-17 expression affecting the immune cell response (Figure 4A). Here, we will report a picture of the mechanisms and the outcomes in different immune cell types involved in the antitumor response. Dll4-triggered Notch activation of CD4+ T cells under Th17 skewing conditions (stimulation with IL-6 and TGF-β) is associated with the production of IL-17 and Th17-related cytokines, IL22 and IL23. Mechanistically, Dll4-stimulation mediates the direct transactivation of IL-17 promoter *via* RBP-Jk activation. In addition, ICN may also bind and transactivate the promoter of retinoic acid-related orphan receptor (Ror-γt), a transcription factor required for Th17 differentiation and the main regulator of IL-17 (163, 164). Overall, this leads to increased IL-17 production and enhanced differentiation of Th17 cell population. Accordingly, Notch signaling blockade significantly reduced IL-17 production, even under Th17 skewing conditions (163). Osborne’s group confirmed that IL-17 is a direct transcriptional target of Notch in Th17 cells and that the differentiation of these cells requires Notch activation (165).

Notch signaling is also involved in promoting an alternative DC phenotype able to stimulate T cells to release IL-17. Indeed, DCs are known to express both Notch receptors and ligands (122). The group of Dallman showed that Jagged1 may trigger DC maturation in an alternative way from the LPS-toll-like receptor signaling (166). Jagged1-conditioned DCs promotes survival, proliferation and increases the suppressive ability of Tregs. Moreover, Jagged1-conditioned DCs produce IL-2 that stimulates CD25+ T cells to produce IL-17 (166).

The activity of Notch signaling in promoting IL-17 expression links Notch to the differentiation of another population of T lymphocytes, γδ T-17 cells, a subset of γδ T cells that shares many of the Th17 phenotypic markers and effector cytokines (IL-17 and IL-22) and has important functions in inflammation and antitumor immunity (168–170).

Notch activation is known to be involved in thymic determination and regulation of the innate function of γδ T-17 cells in thymus and periphery (28) (Figure 4B). Specifically, HES1 induces γδ T-17 cell development and IL-17 production (28). In the thymus, IL-6 stimulates thymic epithelial cells to express Dll4 resulting in Notch activation in γδ T-17 cells (167). In peripheral tissues, such as the intestine and lungs, the high levels of Dll4 expression (171), stimulate a Notch-dependent increase of IL-17 γδ T cells (28).

The only evidence that links Notch activity to the antitumor functions of γδ T-17 in cancer has been reported by Gogoi et al. These authors showed that Notch stimulates the activation and proliferation of peripheral γδ T-17 cells. Notch inhibition, mediated by GSI, reversed this effect and blocked γδ T-17 cell production of cytokines including IFN-γ, TNFα, and IL-17, resulting in the decrease of γδ T-17 cell antitumor cytolytic effect on oral cancer cell lines (29).

## CCL5

The inflammatory chemokine CCL5 and/or its receptor, CCR5, are expressed in various human cancers, including breast cancer, prostate cancer, ovarian and cervical cancer, gastric and colon cancer, melanoma, multiple myeloma, Hodgkin’s lymphoma, and T-acute lymphoblastic leukemia (88, 172–174). Further source of CCL5 in TME may be infiltrating leukocytes, BMSCs, mesenchymal stem cells, or tumor-associated fibroblasts (174).

CCL5 may enhance tumor development in multiple ways: acting on tumor cells by inducing proliferation, invasion, and metastasis, shaping the TME by stimulating the activation of carcinoma-associated fibroblasts (CAFs) and OCLs (in bone-associated cancer or bone metastasis), and shaping the immune infiltrate toward immunosuppression by promoting the apoptosis of cytotoxic CD8+ T cells, the recruitment of TAMs, MDSCs, eosinophils, mastocytes, CD4+ T cells, and T regulatory cells (174).

Notch has been reported to positively regulate CCL5 expression in multiple myeloma-associated BMSCs (173) and in breast cancer (30). As reported above, BMSC-derived CCL5 together with other chemokines results in enhanced myeloma cell viability and migration (173). A recent report demonstrates that the axis CCL5/CCR5 play a key role in a metabolic feedback loop between breast cancer cells and macrophages with important outcomes on immune system infiltrate (30). Cancer cells may have a high rate of glycolysis even in the presence of oxygen; this effect is known as aerobic glycolysis or Warburg effect (175) and results in the production of high levels of lactic acid, which in turn decreases pH in TME that may confer a proliferative advantage to cancer cells. Lactic acid produced by breast cancer cells supports TAM M2 polarization and their production of CCL5 by increasing Notch1 and Jagged2 mRNA and protein expression (30). In return, TAM-derived CCL5 induces breast cancer cell migration, EMT, and promotes aerobic glycolysis *via* AMPK signaling activation, resulting in increased metastatic ability (30).

In conclusion, the crosstalk between lactate, Notch signaling, and CCL5 has several deleterious outcomes: increased metastatic ability and TAM recruitment. In addition, although no direct evidence has been reported, it is conceivable that the increased acidification and decreased glucose availability greatly influence T cell metabolic fitness (176), switching the infiltrated T cell populations from cytotoxic to regulatory. Indeed, lactate, directly and indirectly, affects T cell proliferation and activation; while low glucose levels hamper the activation of effector T cells and induce their apoptosis, thereby favoring the increase of Tregs, that is not reliant on high rates of glucose metabolism (176).

## Notch may Shape the Composition of the Immune Cell Infiltrate by Regulating IL-1β and CCL2

IL-1α and IL-1β are pro-inflammatory cytokines, members of the interleukin 1 family. IL-1α is mainly secreted by macrophages, neutrophils, and endothelial cells in the acute inflammatory response where it collaborates with TNFα to promote systemic inflammation and fever. Notably, both IL-1α and IL-1β are crucial components of the pro-inflammatory secretory profile of senescent cancer cells as detailed below.

IL-1β is predominantly produced by activated macrophages and adipocytes in the TME, although cancer cells may contribute to increasing its levels (31, 177). IL-1β production is a two-step process involving the production of an inactive IL-1β proprotein (pro-IL-1β), followed by its activation induced by caspase-1, a component of the activated multiprotein complex called inflammasome together with the Nod-like (NALP) and apoptosis-associated speck-like (ASC) (178).

IL-1β has a pleiotropic and controversial role in cancer. It is a crucial mediator of the innate immune response, promotes tumor growth, angiogenesis, and metastasis in several tumor types including breast cancer, melanoma, non-small-cell lung carcinoma, and colorectal adenocarcinoma (177).

Breast cancer represents one of the better-studied models and expresses all the members of the IL-1 system, including IL-1α and β, antagonist IL-1Ra, and receptor IL-1R (177, 179, 180).

IL-1β transcription is regulated by Notch in cancer cells, differently from its regulation in the cells of myeloid lineage, where its expression is stimulated by the engagement of toll-like receptors or endogenous danger signals. Studies in breast cancer cells clearly demonstrate that Jagged1, ICN1, and ICN3 are required for IL-1β transcriptional activation occurring at the RBP-Jk DNA binding site at −2,085 from the translation start site (31). Despite this direct transcriptional regulation, Zheng et al. suggest that IL-1β transcription may be triggered by a more complicated mechanism relying on the activation, mediated by phosphorylation, of STAT3 and requires an active Notch1 signaling (181).

Through the regulation of IL-1β, Notch may shape the immune infiltrate at the tumor site, affecting both the innate and the adaptive antitumor immune response. IL-1β is a pleiotropic cytokine and its role in cancer might be context dependent even if this statement is still under discussion (182) since opposite outcomes have been reported ranging from cancer protection to cancer progression.

A protective role for IL-1β is reported in models of colon cancer associated to colitis, possibly due to the concomitant production of IL-18, relevant for intestine healing (183), or in a model of epithelial skin carcinogenesis, where the inflammasome adapter ASC may play a protective role in keratinocytes. By contrast, in the same model of skin carcinogenesis, ASC plays as a tumor promoter in myeloid cells (184), and IL-1β and inflammasome are crucial for mesothelioma development (185) and murine mammary carcinoma progression mediated by myeloid recruitment (186).

The pivotal role or Notch in the modulation of immune cells infiltrating the TME has been mainly studied in breast cancer. Notch regulates the recruitment of TAMs in two different ways. It promotes the expression of IL-1β and CCL2 and supports monocyte adhesion to blood vessel endothelium in synergy with CCL2 that promotes chemotaxis and extravasation (31).

Tumor-associated macrophage infiltration is associated with poor prognosis (187) and sustains a cytokine milieu abounding of TGF-β, IL-1β, and CCL2 that collaborate to promote monocytes recruitment and promotes an immunosuppressive TME. The underlying mechanism is complex and involves a synergy of tumor cells and TAMs. These cells secrete TGF-β that promotes Jagged1 expression in tumor cells. Tumor-derived Jagged, in turn, boosts the expression of IL-1β and CCL2 in the same tumor cells and in TAMs (32). In addition, Notch activation in tumor cells potentiates TGF-β signaling by promoting the secretion of the urokinase-type plasminogen activator, that allows the maturation of the immature form of TGF-β released by TAMs and further sensitize tumor cells to TGF-β through the upregulation of TGFβR1 (31).

A further reinforcement of an immunosuppressive TME in breast cancer may be induced by high levels of IL-1β. Indeed, high IL-1β levels have been reported to be associated with impaired activation of CD8+ T cells and systemic expansion and polarization of immunosuppressive neutrophils (32, 188). The expansion of this population seems to be owed to IL-1β ability to activate IL-17-producing γδ T cells responsible for increased systemic levels of G-CSF, a cytokine known for its role in granulopoiesis (188).

The relevance of the interplay between Notch and IL-1β is strengthened by the evidence that effective IL-1β and CCL2 antagonists are currently in clinical review to treat benign inflammatory disease, and their transition to the cancer clinic has been proposed (31).

The nasty outcomes of the cooperation between Notch and IL-1β in cancer may be potentiated by body metabolism, specifically by leptin, a hormone whose levels are significantly increased with obesity. Also in this case, most studies focus on breast cancer, where leptin acts as a positive regulator of Notch expression and activation in estrogen responsive and triple-negative breast cancer (TNBC) cells through canonic JAK2/STAT, MAPK1/2K 1/2, and PI3K/AKT, and non-canonic signaling pathways JNK and p38 MAP kinases (189, 190).

Here, besides the reported Notch-mediated increases of the expression of IL-1β, VEGF, and VEGFR2, the authors demonstrate that IL-1β signaling is required for the positive regulation of Notch receptors induced by leptin (190); moreover, beside the immunosuppressive effect of Notch signaling reported above, Notch, IL-1β and leptin crosstalk outcome mediates other key features including cell proliferation, survival, migration, and angiogenesis in breast cancer (190) and likely in other tumors including pancreatic and endometrial cancer (189, 191).

Inflammatory TME plays a key role in the self-renewal of cancer stem cells (CSCs). In particular, the interplay between Notch and IL-1β in TME is reported also to affect CSCs in TNBC resulting in increasing their self-renewal. Indeed, metastatic TNBC cells in the brain express high levels of IL-1β that stimulates the neighboring astrocytes to express Jagged1. This in turn triggers Notch signaling upregulation in CSCs enhancing their self-renewal (192).

Concerning CCL2, its production from different cell types, such as fibroblasts, OBLs, endothelial cells, and smooth muscle cells, is thought to promote cancer growth and metastasis (193). Notch pathway has been frequently reported to positively regulate CCL2 expression (31), although this effect seems to be cell type specific, indeed in experimental liver fibrosis and patients with acute-on-chronic liver failure Dll4 is inversely associated to CCL2 (194) and in the melanoma cell line M624 silencing of the Notch coactivator MAML1 results in CCL2 mRNA and protein upregulation (195). The outcome of Notch-mediated positive regulation of CCL2 in breast cancer cells in synergy with IL-1β has already been described (31). Further noteworthy outcomes of the Notch/CCL2 axis are important in the nasty communication between tumor cells and BMSCs in the primary tumor site and in the metastatic one. Multiple myeloma cells primarily reside at the BM, where they get an advantage and induce BMSCs to a pro-tumor behavior in different ways, including conveying extracellular vesicles containing different stimuli among which miRNAs. Tumor-derived miR-146a may induce the activation of Notch1 in BMSCs stimulating them to secrete CCL2 and several cytokines including IL-8, IL-6, CXCL1, IP-10, and CCL5 that enhance myeloma cell viability and migration (173). An elegant study by Yumimoto et al. explored the mechanisms of cancer metastasis, showing that lung metastases are promoted by BMSCs migrated to the lungs, and identified a trigger of the metastatic process in the low expression of the tumor suppressor FBXW7 in TME, a condition associated with poor prognosis in breast cancer patients (33). The FBXW7 role is based on its ability to downregulate Notch signaling since it mediates a key step in the degradation of ICN (and other oncogenes), acting as substrate recognition component in the SCF-type ubiquitin ligase complex. *In vivo* experiments showed that loss of FBXW7 in BMSCs results in the accumulation of ICN1, that in turn promotes the secretion of CCL2. CCL2 is a chemotactic stimulus for the recruitment of Mo-MDSCs and macrophages that, in turn, induce the metastatic site to sustain the growth of tumor cells that have already colonized the lungs (33).

## Notch and IFNγ Cooperate to Shape the Immune Cell Landscape

IFN-γ is a key promoter of macrophage activation and induction of MHC-II expression. The most important sources of IFN-γ are cells of T lineage including CD8+ T cells and Th1 cells, NK and NK T cells belonging to the adaptive or the innate immune system (196).

The antitumor activity of IFN-γ stems from several distinct mechanisms:

(1) tumor-directed anti-proliferative and pro-apoptotic actions, based on STAT1 activation and the expression of, respectively, cell cycle inhibitors such as p21 and p27, or apoptotic mediators including caspase-1 or Fas and Fas ligand (197);
(2) inhibition of angiogenesis, indirectly induced by a family of interferon-induced chemokines with potent angiostatic actions, i.e., IP-10, Mig, and I-TAC (197);
(3) potentiation of the killing immunological functions, including (a) development of antitumor adaptive immune response mediated by (i) IFN-γ ability to direct the appropriate Th1/Th2 balance by stimulating Th0 cell polarization toward Th1 and inhibiting Th2 cell differentiation; (ii) IFN-γ-mediated stimulation of MHC-I expression by tumor cells with the consequent increase of tumor-antigen presentation; (iii) activation of the tumor cell killing mediated by T CD8+ cells; (b) the activation of the host antitumor innate immune response, mediated by macrophages and NK cells (197).

IFN-γ has been reported to be a direct transcriptional target of Notch; a study performed on Th1 cells demonstrated that Notch is recruited to the RBP-Jk-binding elements at an enhancer site of the IFN-γ gene (198). We will briefly describe the role played by Notch in regulating the important activity of IFN-γ in the regulation of antitumor innate and adaptive immune response by examining the outcome on the key cell types involved: DCs, NKs, CD8+, and CD4+ T cells.

Concerning DCs, Notch and IFN-γ collaborate to promote their maturation and the ability to activate the different T cell subsets. Notch positively regulates DC maturation; indeed, Notch ligand Jagged1 induces the upregulation of maturation markers, IL-12 production, and DC ability to promote T cell proliferation and maturation in effector T cell as demonstrated by IFN-γ production (34). Moreover, the CD80/CD86 triggered upregulation of IL-6 secretion, necessary for full T cell activation, occurs through the collaboration of Notch and PI3K signaling (199). Finally, Notch signaling increases the expression of MHC complexes in DCs, necessary for T cell activation.

A great part of the role of Notch signaling in antitumor response is due to DC ability to activate Notch signaling in interacting lymphocytes through the expression of Notch ligands. Among the physiological stimuli promoting the expression of Notch ligands in DCs, GM-CSF and CpG DNA have been reported to increase Jagged2 expression (35), while LPS induced Jagged1 and Dll4 production (200). DC-derived Notch ligands participate in the instruction of T helper cells to commit to the Th1 (201), Th2 (122), Th17 (163), or Treg (202) lineage.

Recent studies showed that NK cells can be activated by DCs and macrophages (35, 203, 204) and that Jagged and Dll ligands can promote the development or activation of NK cells *in vitro* (205, 206). Kijima and colleagues (35) confirmed *in vivo* that DCs can increase NK cell cytotoxicity by stimulating the activation of Notch2 on NK cells through the ligand Jagged2, whose expression may be stimulated by GM-CSF and CpG DNA. Importantly, Jagged2 stimulated NK cells to increase IFN-γ secretion and cytolytic activity resulting in decreased tumor burden in a murine lymphoma model (35).

The generation of cytotoxic CD8+ T cells is essential for tumor control. DCs provide key signals to induce the priming and activation of CD8+ T cell. Notch pathway has an important role in both these processes in human CD8+ T cells. Indeed, the activation of Notch2 signaling on CD8+ T cells, mediated by Dll4 or Jagged1 expressed by DCs, is required for the activation and proliferation of human CD8+ T cells and for the release of effector cytokines including IFN-γ, along with TNFα, perforin, and granzyme B (36, 37, 200, 207).

Auderset et al. reported that Notch pathway is involved also in the differentiation and activity of the Th1 subset of CD4+ T cells (38). Specifically, Notch signaling mediated by Notch1 and/or Notch2 induces IFN-γ secretion by CD4+ Th1 cells. Interestingly, the involvement of Notch signaling seems to provide a possible alternative stimulus for Th1 cell differentiation in the absence of the skewing cytokine IL-12 as demonstrated by RBP-Jk ablation or in mice expressing a dominant negative MAML transgene (208). Indeed, upon LPS stimulation CD8− DC subtype induces MyD88-dependent expression of Dll4, which in turn may engage Notch on Th1 cells inducing IFN-γ expression, differently from LPS effect on CD8+ DCs that results in increased IL-12 expression (209).

In contrast to the collaboration of IFN-γ and Notch in promoting Th1 cell differentiation, IFN-γ and Notch play antithetic roles in Th2 cell polarization. IFN-γ may potentiate the Th1 shift by an inhibitory effect on Th2 cell response, while essentially Notch antagonizes IFN-γ-induced inhibition of Th2 cell differentiation. A confirmation of this role comes from the evidence that upon IFN-γ neutralization Notch activation becomes dispensable (198).

## VEGF and Notch Cooperate to Induce Tumor Angiogenesis and Evasion from the Immune System

VEGF regulates different aspects of tumor progression including angiogenesis, but although its role in cancer progression is much wider. Recently, an immunosuppressive function of VEGF has emerged that protects cancer cells from the increased recruitment of immune cells at the tumor site promoted by neoangiogenesis. Indeed, if tumor vasculature is key in providing tumor cells with oxygen, nutrients, and glucose, along with an escape to enter blood circulation allowing tumor metastasis, it also maximizes the exposure of tumor cells to the antitumor activity of immune cell populations. Thereby, we will detail the interaction of VEGF with Notch signaling in regulating these important aspects of tumor progression.

VEGF is known as a major pro-angiogenic signaling pathway involved in developmental, physiological, and tumor-associated angiogenesis. VEGF family consists of six ligands (VEGF-A, -B, -C, -D, -E, and placental growth factor) with different affinity to the four VEGF receptors: VEGFR1–2–3 and neuropilin 1 (210). This family regulates almost all steps of new vessels formation including sprouting and intussusceptive angiogenesis, vessel maturation, and differentiation into arterioles, venules, and capillaries (211).

**Figure 5:**
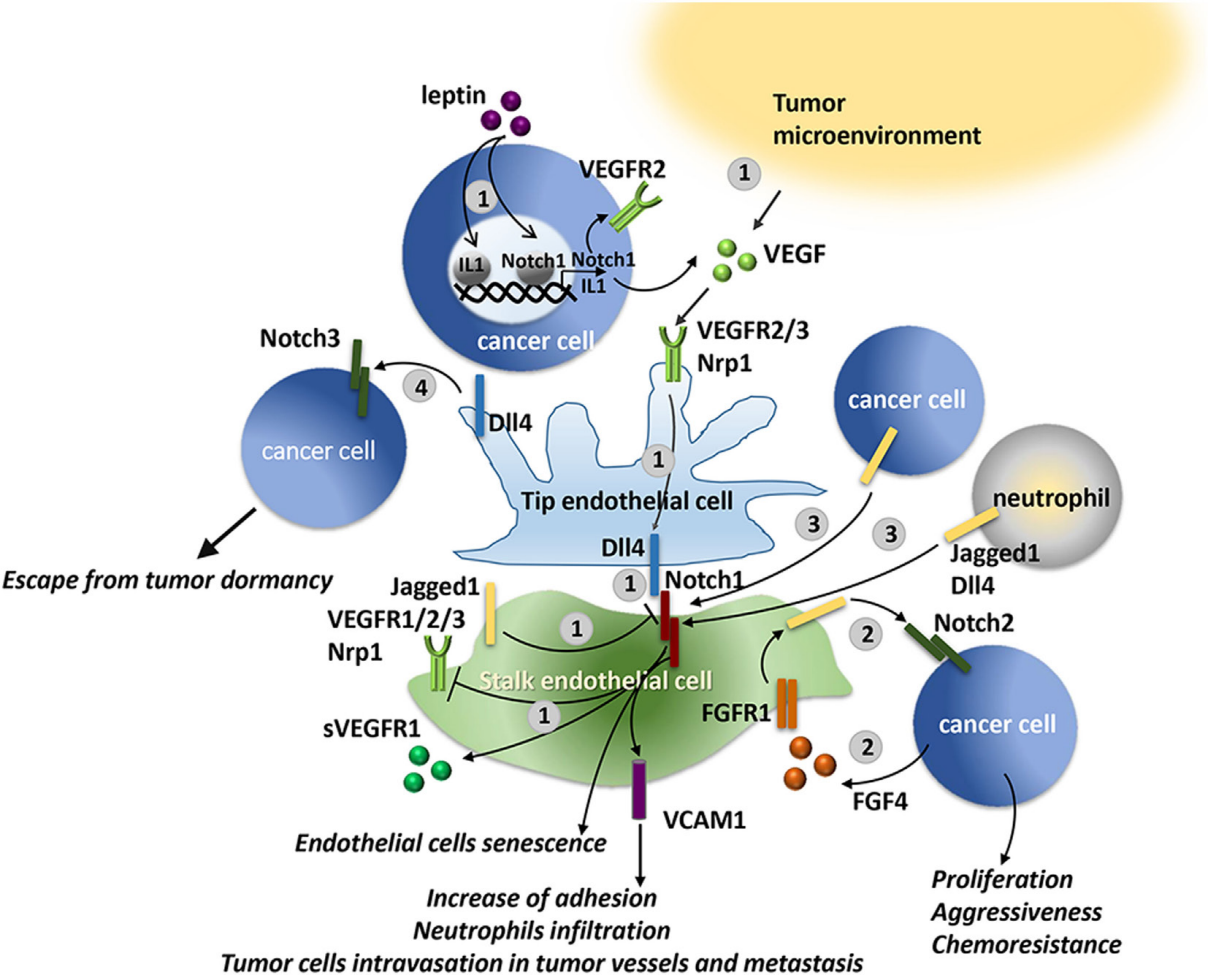
VEGF and Notch interaction in physiological and tumor angiogenesis. Notch controls tip/stalk endothelial cells balance in tight cooperation with VEGF. 1. VEGF may be produced by cancer cells, for example, in breast cancer cells, leptin through IL-1 and Notch1, induces VEGF/VEGFR-2 upregulation (190), or by the neighboring cells of the tumor microenvironment (TME). VEGF engages VEGFR2/3 and neuropilin 1 (Nrp1) receptors on endothelial cells, activating a signaling that promotes Dll4 expression and determines the differentiation toward a tip cell type. Dll4 expressed on tip cells triggers Notch signaling in the neighboring cells suppressing the tip cell phenotype and inducing the differentiation in stalk cell. Notch activation in stalk cells either decreases VEGFR1/2/3 expression or stimulates antiangiogenic soluble splice variant of VEGFR1 (sVEGFR1) leading to a reduced sensitivity to VEGF (213). Oppositely to Dll4, Jagged1 expressed by stalk cells antagonizes Dll4-mediated Notch signaling in stalk cells, thereby increasing tip cell number and sprouting (214). 2. B-cell lymphoma cells release FGF4 that engages FGFR1 on the endothelial cells. FGFR1 activation induces Jagged1 upregulation that, in turn, triggers Notch2 signaling in lymphoma cells promoting their proliferation, tumor aggressiveness, and chemoresistance (215). 3. Notch1 signaling in endothelial cells may be activated by tumor-derived ligands, i.e., Jagged1 and Dll4 expressed by lung carcinoma and melanoma cells, or by Jagged1 expressed by neutrophils. Notch1 activation promotes endothelial cell senescence and expression of the adhesion molecule, VCAM1, that promotes neutrophil infiltration and tumor cell metastasis (216). 4. In models of tumor dormancy of colorectal carcinoma and T cell acute lymphoblastic leukemia (T-ALL) it was shown that Dll4 expressed on tumor endothelial cells can trigger Notch3 pathway activation in tumor cells conferring tumorigenic properties to the dormant cells (217).

Aberrant sprouting angiogenesis is characteristic of tumor vasculature. VEGF and Notch cooperate to control the sprouting of new vessels by tightly regulating the balance between tip and stalk cells (212) as detailed in Figure 5. Thereby, it is evident that increased levels of Notch signaling or VEGF levels in cancer may locally alter the vasculature.

Almost all tumors express VEGF and high intratumor and serum levels of this cytokine are associated with poor prognosis in cancer patients (218, 219). The release of VEGF by tumor cells is activated by distinct microenvironmental cues, including hypoxia and inflammatory cytokines, or by deregulated oncogenes and pathways (220). The outcome is an “angiogenic switch” with the formation of new vasculature around the tumor, that promotes its growth, invasion, and metastasis (221). Moreover, new blood vessels have an immunosuppressive effect by expressing inhibitory molecules such as programmed cell death ligand 1 (PDL1) and PDL2, indoleamine 2,3-dioxygenase, the adhesion molecule CD31 that may inhibit T cell activation and immunosuppressive cytokines such as IL–10, IL-6, and TGF-β (222). Also, tumor vasculature and high VEGF levels may shape the TME by controlling immune cells extravasation, i.e., promoting the migration of Treg cells while hampering the infiltration of effector T cells and thereby favoring an immunosuppressive cytokine milieu (222).

Tumor vasculature is triggered by the crosstalk between VEGF and Notch signaling within the tumor-associated endothelial cell (213). High levels of VEGF in TME, derived from the tumor, endothelial, stromal cells, and immune cells such as macrophages and Tregs, induce an aberrant activation of Notch signaling in tumor-associated endothelial cells that promotes the formation of new altered vessels by replicating a dysregulated version of physiologic angiogenesis (213, 223, 224).

Studies on mouse tumor models confirmed the involvement of VEGF/Notch axis in the formation of tumor endothelium that supports tumor growth. As reported in Figure 5, tumor-derived VEGF induces Dll4 expression in tumor vessels resulting in increased number of stalk cells, characterized by high Notch activity, at the expense of the tip cells. As a consequence, the formed vasculature displays a reduced density, but enhanced vessel diameter and perfusion, and thereby support tumor growth (225, 226). Conversely, as expected, the inhibition of Notch in mouse models of glioma, lymphoma, fibrosarcoma, colorectal, lung, and mammary gland tumors, by systematic retroviral delivery of soluble blocking version of Dll4 or anti-Dll4 antibodies increased VEGFR expression in endothelial cells, vessel branching and density of the tumor vasculature that led to reduced tumor growth due to poor perfusion of tumor vessels and increased hypoxia (226, 227). Consistently, a Notch1 decoy that inhibits the interaction Notch–Dll caused a hypersprouting phenotype, stimulated dysfunctional tumor angiogenesis, and hampered tumor growth in xenograft mouse models of mammary, pancreatic, lung tumors, and melanoma (228). The relevance of the cooperation between VEGF and Dll4-mediated Notch signaling is highlighted by the fact that blocking of Notch signaling through Dll4 neutralizing antibody increases sensitivity to anti-VEGF therapy and reduces tumor growth (225, 227).

High expression of Jagged1 in tumor endothelium destabilizes the tip/stalk balance resulting in a hybrid tip/stalk phenotype leading to enhanced sprouting angiogenesis that promotes tumor growth (214). In accordance, it was reported that in ovarian cancer murine model targeting Jagged1 in tumor-associated stroma mainly composed of endothelial cells and fibroblasts, led to reduced tumor microvessel density and tumor growth (229). Consistently, Cao et al. demonstrated that B-cell lymphoma cells through FGF4/FGFR1 signaling upregulated Jagged1 on adjacent endothelial cells; in turn, Jagged1 activated Notch2 signaling in the lymphoma cells promoting tumor aggressiveness and chemoresistance (215). In line with this evidence, in models of tumor dormancy of colorectal carcinoma and T-ALL, Indraccolo et al. demonstrated that endothelial Dll4 regulated Notch 3 signaling in tumor cells allowing the escape from tumor dormancy (217).

Besides the aberrant Jagged1 expression in tumor-associated endothelial cells, also tumor cells overexpress Jagged1 that plays role in tumor sprouting angiogenesis and in the release of pro-inflammatory chemokines by endothelial cells. Zeng et al. showed that in head and neck squamous cell carcinoma tumor-derived Jagged1 triggered the activation of Notch in neighboring endothelial cells, stimulated the sprouting of capillary-like formations and significantly increased neovascularization and tumor growth *in vivo* (230). Consistently, it was demonstrated that Jagged1 and Dll4 expressed by lung carcinoma and melanoma cells and Jagged1 expressed by neutrophils triggers Notch1 activation in endothelial cells inducing their senescence along with the expression of chemokines and the adhesion molecule VCAM1, that favor neutrophil infiltration, tumor cell intravasation in tumor vessels and metastasis (216).

Kitajewski’s group showed that in mammary gland tumor murine model, in which Jagged1 tumor expression was upregulated by ectopic expression of FGF4, Notch inhibition through Notch1 decoy disrupted tumor angiogenesis and delayed the growth of murine Mm5MT xenografts (231). The same group later showed that the interplay between Jagged1 and VEGF promotes tumor endothelial branching along with vascular mural maturation that requires the involvement of Jagged1 (228). Using Notch1 decoy which specifically inhibits Jagged-class mediated Notch activation, the authors showed that Jagged ligands positively regulate angiogenesis by suppressing sVEGFR1 and promoting the interaction between mural cells and endothelial cells (228). Thereby, selective Jagged blockade using a Notch decoy increases sVEGFR1 levels, suppressing sprouting and perfusion, and disrupts pericyte and vascular smooth muscle cell coverage in tumor endothelium of mouse models of mammary, pancreatic, lung tumors, and melanoma, resulting in inhibited tumor growth (228).

A recent study on invasive mammary micropapillary carcinomas hypothesized also a cooperation of VEGF and Notch in tumor lymphangiogenesis. Here, the active form of Notch1 is expressed in extra-tumoral lymphatic endothelial cells together with a receptor of VEGF-C, VEGFR3, involved in lymphatic endothelial cell proliferation, tumor lymphatic invasion, and tumor metastasis (232).

So far, we have described a cooperative activity where VEGF released in TME positively stimulates tumor angiogenesis by regulating Notch signaling in endothelial cells. But, Notch activation in tumor cells and neighboring cells may positively regulate the levels of VEGF released in the TME, with a consequent stimulation of angiogenesis, tumor, and stromal cells.

In breast cancer cells, Notch signaling is necessary for leptin-induced expression of VEGF and VEGFR2 (as detailed above) suggesting that Notch is a downstream mediator of leptin-mediated regulation of breast cancer cell growth and tumor angiogenesis (190). Consistently, Notch1 blockade results in downregulated secretion of VEGF associated with a reduction of tumor angiogenesis and tumor cell invasive abilities (233). In pancreatic tumor cells, exogenous Jagged-1 expression induced VEGF secretion and increased the invasive phenotype of pancreatic cancer cells (234). The Notch–VEGF axis is also exploited by tumor cells to shape the surrounding BMSCs and activate their angiogenic effect as demonstrated by two studies on multiple myeloma. These groups demonstrate that Jagged2, overexpressed by myeloma cells, induces Notch activation in BMSCs, which in turn activates VEGF secretion. Secreted VEGF promotes angiogenesis and acts as a growth factor for myeloma cell stimulating its proliferation (235, 236).

Finally, we must mention that Notch and VEGF signaling cooperated in promoting the “vascular niche” formation necessary for CSC expansion. This aspect has been mainly explored in CNS tumors. Studies on glioblastoma multiforme (GBM) confirmed that Notch plays a role in endothelial control of CSCs. Indeed, the inhibition of Notch signaling blocks GBM CSC self-renewal by decreasing the number of endothelial cells. In turn, the CSC niche promotes angiogenesis, by CSC-mediated release of VEGF or through pro-angiogenic cytokines produced by tumor-infiltrating lymphocytes, such as Th17 cells, and macrophages (237).

As anticipated VEGF functions in cancer are not restricted to tumor angiogenesis. Indeed, VEGF produced in the TME sustains tumor progression also playing an immunosuppressive role by regulating various types of immune cells, such as DCs, T cells, macrophages, and MDSCs (238). Since many kinds of immune cells express VEGF receptors, functions of these cells can be regulated by tumor-derived VEGF. For instance, through the activation of VEGFR1, VEGF inhibits the maturation and activation of DCs with a consequent reduced CD8+ T cell response against tumors such as colorectal, gastric, lung, and breast cancer (239–243).

VEGF contributes to suppress antitumor immune response interfering with CD4+/CD8+ T cell development and differentiation (244). Tumor-derived VEGF facilitates the infiltration and proliferation of Tregs by engaging VEGF receptors, localized on the surface of Tregs (245, 246). It should be noted that infiltrated Tregs recruited to hypoxic areas may also contribute to increasing VEGF levels in the TME sustaining also tumor angiogenesis (223).

VEGF may affect the myeloid immune cell landscape. Indeed, VEGF may attract immature myeloid cells from the BM into the tumor where, in cooperation with other factors, such as IL-10 and TGF-β, induces these precursors to differentiate into M2 macrophages (247) or may also directly recruit macrophages to the tumor site (248). Tumor-derived VEGF stimulates MDSCs expansion through binding to VEGRF1; consistently, bevacizumab treatment of patients with renal cell cancer showed decreased the number of MDSCs in the peripheral blood (249).

Although the respective functions of Notch and VEGF as immunomodulators are quite well studied along with their interplay in directing tumor angiogenesis, little is known about their interaction and collaboration in tumor immune escape. A study performed by Huang et al. confirmed the hypothesis that their cooperation may contribute to the tumor evasion of immune cell surveillance by demonstrating that Notch-VEGF crosstalk affects the immunosuppressive function of T-cells (39). In a mouse model, the chronic infusion of VEGF, that mimicked the pathophysiologic VEGF concentrations observed in patients with advanced-stage cancer, reduced the levels of Dll1 and Dll4 observed in BM cells (39). Low levels of Dll ligands resulted in the suppression of T cell function, observed as a decrease of the T/B cells ratio in the spleen. Conversely, the selective activation of Dll1-mediated Notch signaling in BM precursors in tumor-bearing mice resulted in the increase of tumor-infiltrating T cells and enhanced activation of Th1-type IFNγ-producing T cells, resulting in tumor growth inhibition.

Finally, VEGF may promote immunosuppression also by contributing to activate the immune checkpoints effector, PD-1. PD-1 is a T cell surface receptor that limits T cell response intensity to avoid autoimmunity. PD-1 regulates two different steps in T cell activation. During DC-mediated activation of T cells, PD-1 engaged by its ligand, PDL1, expressed on DCs may promote apoptosis in effector T-cells and survival in regulatory T cells, resulting in self-tolerance and suppression of T cell cytotoxic activity. Thereby, high levels of PD-1 are involved in immune evasion *via* induction of T cell exhaustion and tolerance for tumor antigens (250). Tumor-derived VEGF has been shown to enhance expression of PD-1 on activated CD8+ cells of colon carcinoma murine models through VEGFR2 signaling, which could be reverted by antiangiogenic agents targeting VEGF-A–VEGFR (251). In addition, VEGF may also induce the expression of PDL1 on tumor-associated myeloid DCs, thus impairing DC-mediated T-cell stimulation (252).

Although a direct collaboration of VEGF with Notch has not been reported yet in the regulation of immune checkpoint, the two pathways clearly act synergistically, since also Notch signaling may play a role in T cell exhaustion. As a matter of fact, Notch binds to PD-1 promoter stimulating its transcription in activated CD8+ T cells. Thereby, Notch signaling inhibition, induced by DAPT or SAHM1, affects PD-1 expression, and restores the function of effector T cell (253).

Overall, shreds of evidence presented here indicate that the interaction between Notch and VEGF influences various cell type in TME promoting tumor cell growth, angiogenesis, metastasis, and escape from the antitumor immune response. Future works are needed to further elucidate a role of the crosstalk between Notch and VEGF with particular attention to tumor-associated immune suppression since uncoupling this interaction may increase the potential of immunotherapy to circumvent the evasion of antitumor immune response.

## Notch Regulates the Cytokine Profile Associated to Cancer Cellular Senescence and Its Outcome on Innate and Adaptive Immune System

The evidence reported in the previous paragraphs indicates that a complex connection exists between Notch signaling levels in tumor and healthy cells populating the TME, the cytokine milieu and different outcomes on tumor progression. Recently, it was reported that Notch signaling ability to control the profile of cytokines secreted by tumor cells may be involved also in cancer cellular senescence.

Cancer-associated cellular stress events, such as DNA damage or activation of oncogenes, may result in cellular senescence. Cellular senescence is a process that leads cells to a permanent proliferative arrest during aging, embryogenesis, and cancer development.

Senescent cancer cells do not proliferate, and as such they do not contribute to the increase in tumor burden, but they may remain metabolically active and secrete proteins with either tumor-suppressing or tumor-promoting activities (254, 255), collectively reported as senescence-associated secretory profile (SASP). SASP is composed of inflammatory cytokines such as TGF-β, IL-1, IL-6, IL-8, growth factors, including VEGF and IGF1, and MMPs similar to those involved in early stage inflammation (255).

On the whole, the phenomenon of senescence may have beneficial effects for the host, such as terminal arrest of cancer cell proliferation and/or its clearance by immune cells recruited through the released cytokines, but often detrimental outcomes may prevail since the secreted senescence-associated cytokines feed non-senescent neighbors inducing their growth or creating an immunosuppressive environment that hampers the antitumor activity of the immune system, resulting in unchecked tumor progression.

**Figure 6:**
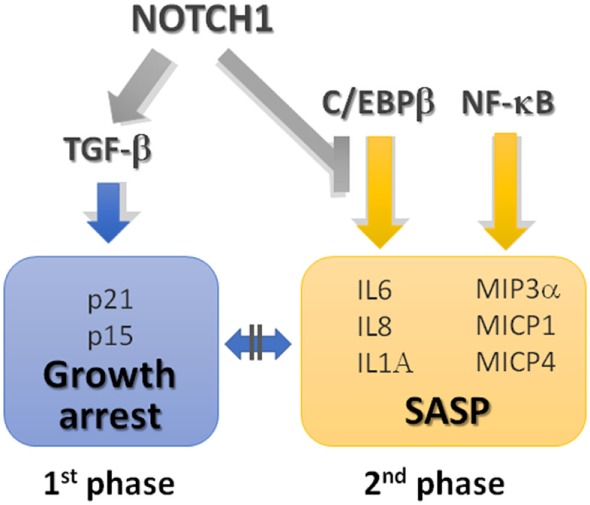
Notch signaling regulates cancer cell senescence and the associated secretome profile. Notch activity is dynamically regulated during the acquisition of the full cell senescence. Through an elegant work on cancer models of oncogene-induced senescence (OIS), Notch-induced senescence (NIS), and DNA damage senescence induced in human diploid fibroblasts, and confirmed in *in vitro* models of pancreatic and liver cancer, Hoare et al. demonstrated that the first phase of senescence is characterized by the activation of Notch with the consequent promotion of transforming growth factor-β (TGF-β) expression and the inhibition of transcriptional activity of C/EBPβ transcriptional activity. This is unleashed with Notch downregulation during full senescence acquisition resulting in the expression of pro-inflammatory senescence-associated secretory profile (SASP) (256). See text for details.

Notch activity plays a leading role in cancer cell senescence, primarily through the isoforms, Notch1, Notch3, and the ligand Jagged1 (256, 257). Notch1 involvement has been studied extensively and therefore its role is better defined. Several reports associate Notch1 activity with the onset of senescence in cancer, but the temporal regulation is complicated by the sequence of two distinct phases, necessary for the full acquisition of senescence, and differently regulated by Notch activity. *In vitro* and *in vivo* experiments from Hoare et al. (256) show that Notch1 activity is dynamically upregulated during the first phase of induction in both oncogene-induced senescence (OIS) and DNA damage-induced senescence (DDIS) and returns to basal levels when senescence is completely established (Figure 6). These two phases, characterized, respectively, by high and low levels of Notch activity, also display two distinct secretomes: the first characterized by anti-inflammatory cytokines and the second by pro-inflammatory cytokines and matrix-modifying enzymes (i.e., MMP1, 3, 10). The mechanism underlying the switch between the two phases involves the interplay between Notch1 and CCAAT/enhancer-binding protein β (C/EBPβ) (256), as depicted in Figure 6. In this first phase, high Notch signaling inhibits C/EBPβ transcriptional activity and promotes the secretion of TGF-β. This cytokine mediates immunosuppression along with the growth arrest observed in senescence, in part through the induction of p15 and p21. Upon full senescence achievement, the decrease of Notch activity releases the transcriptional activity of C/EBPβ that, together with NF-κB, coordinates the secretion of pro-inflammatory SASP including IL-6, IL-8, and the master senescence regulator IL-1A (255).

While OIS and DDIS require a biphasic modulation of Notch signaling, tumor cells showing steady high Notch signaling levels are characterized by a different form of senescence, known as Notch-induced senescence (NIS). NIS produces an unusual immunosuppressive cytokine profile characterized by increased TGF-β, growth arrest, and inhibition of pro-inflammatory cytokines (69). Accordingly, Kagawa et al. (258) reported evidence that the ectopic expression of ICN1 induced cellular senescence by inhibiting cell proliferation, finally resulting in irreversible cell-cycle arrest in G0/G1 phase, through the increased expression of p16INK4A and p21 and Rb dephosphorylation. Similarly, Notch3 increased expression in replicative senescence and in DDIS was associated to increased p21 (257).

In addition, the lack of inflammatory secretome associated to NIS was shown to affect *in vivo* lymphocyte recruitment at the tumor site, at least in part by hampering lymphocyte adhesion to endothelial cells and preventing extravasation (256). This is important, since the local infiltration of innate and adaptive immune system is key to the clearance of senescent cancer cells. Several evidences confirm that Notch signaling antagonizes the formation of an immune-stimulating microenvironment associated to senescence. Indeed, Kang et al. (259) recently reported that inhibition of Notch activity, obtained through the use of DN-MAML, results in: (1) senescent phenotype with the release of immune-stimulating cytokines and chemokines that favor the formation of an immunostimulatory microenvironment, including IL-8, IL-1α, IL-1β (260), IFN-γ, IFN-β, CCL2 (195), CCL5, CXCL9, CXCL10, and CXCL11, CCL18; (2) the tumor infiltration by immune cells, such as DCs, NKs, and CD8+ T cells, in *in vivo* syngeneic animal models.

Another important key feature regulated by Notch signaling is the transmission of senescence from the senescent cancer cells to the neighboring non-senescent ones, caused by two distinct mechanisms: secreted cytokines or cell–cell contact.

The first mechanism involves Notch1-driven TGF-β and induces senescence in a paracrine manner, at least in part by promoting of the expression of the cyclin-dependent-kinase inhibitors p15 and p21 in neighboring cells. The second mechanism involves a cell–cell communication mediated by Notch signaling. Notch activation occurring during OIS or NIS results in increased Jagged1 expression. Jagged1 expressed by senescent cancer cells acts as a master mediator of senescence-lateral induction by activating Notch receptors expressed by neighboring cells (256).

The evidence that many of the Notch target genes encoding for cytokines, i.e., IL-1β, IL-6, IL-8, CCL2, and CCL5 (31, 33, 173, 195), above reported in different tumor settings are negatively regulated by Notch during cancer cell senescence underlines that Notch signaling outcome is highly context dependent.

Senescence may be transmitted to nearby cancer cells, but also to the healthy cells of the surrounding TME. Indeed, Procopio et al. (261) recently uncovered the diffusion of senescence from cancer cells to CAFs through a different mechanism associated with reduced levels of the RBP-Jk transcription factor. RBP-Jk down-modulation is exhibited by CAFs derived from different tumors, including skin squamous cell carcinoma (262), head/neck (263), breast (264), and lung (265) cancers, in comparison to normal fibroblasts. RBP-Jk knockdown in primary fibroblasts from dermis, oral mucosa, breast, and lung, induces cellular senescence, and increases the pro-inflammatory cytokines, such as IL-6. In this scenario, Notch role appears to be further complicated by p53. RBP-Jk acts as a repressor for p53, with which shares DNA binding sites on regulative regions of senescence-associated target genes, including p21, and possibly inflammatory cytokines, such as IL-6. Low levels of RBP-Jk allow p53-mediated transcription and, additionally, the repressor-like activity of RBP-Jk may be unleashed by Notch1 activation, also induced by nearby Jagged-bearing cells (261).

Although several facets remain to be elucidated, including the interplay of Notch activity with other signaling pathways involved in the regulation of senescence, Notch activity in cancer cell senescence is crucial in diffusing senescence from cancer cells to other malignant cells and to non-cancerous cells, and in providing a senescence-associated anti-inflammatory secretome that contributes to hamper the antitumor immune response. Overall, the evidence indicates that a Notch-directed therapeutic approach is a unique opportunity to re-establish the local antitumor response of the immune system.

## Conclusion

Tumor cells are characterized by their ability to shape the surrounding microenvironment, altering the behavior of neighboring normal cells to sustain tumor growth, drug resistance, neoangiogenesis, and bone destruction. In addition, malignant cells cause an immune imbalance in the TME, impairing the functions of cells involved in innate and adaptive immune response, leading to immunesuppression, and promoting inflammation. Overall, these processes contribute to determine the fatal outcome of several malignancies.

In this context, increasing attention has recently been paid to the cytokine network in the TME and to its ability to redefine immune cells differentiation, as testified by increasing number of clinical trials involving drugs or monoclonal antibodies targeting cytokines or their receptors (see Table 2).

**Table 2:** Overview of the last clinical trials that targets cancer-altered cytokines.

Cytokine	Cancer type	Clinical trial phase	Drug	ClinicalTrials.gov Identifier or reference^a^
TGFβ	Prostate cancer	Phase 2	Galunisertib	NCT02452008
TGFβ	Advanced metastatic carcinoma	Phase 1	Galunisertib	NCT02423343
TGFβ	Hepatocellular carcinoma	Phase 2	Galunisertib	NCT01246986 (266)
TGFβ	Metastatic cancer, pancreatic cancer	Phase 1/2	Galunisertib	NCT01373164 (267)
IL-6	Patients with hormone refractory prostate cancer	Phase 2	CNTO 328 (anti IL-6 monoclonal antibody) alone or in combination with n combination with mitoxantrone	NCT00433446, NCT00385827 (268)
IL-6	Patients with unresectable or metastatic kidney cancer	Phase 2	Siltuximab (CNTO 328, anti IL-6 monoclonal antibody)	NCT00311545
IL-6R	Subjects with metastatic HER2 positive breast cancer	Phase 1	Anti-IL-6R monoclonal antibody tocilizumab in combination with trastuzumab and pertuzumab in	NCT03135171 (269)
IL-6	Subjects with relapsed or refractory multiple myeloma	Phase 2	CNTO 328 (anti-IL-6 monoclonal antibody) in combination with bortezomib	NCT00401843 (270)
IL-6	Subjects with newly diagnosed, previously untreated multiple myeloma requiring systemic chemotherapy	Phase 1b/2	Siltuximab (CNTO 328) with lenalidomide, bortezomib, dexamethasone	NCT01531998 (271)
CXCR4	Acute myeloid leukemia	Phase 1/2	Plerixafor	NCT01435343 (272)
CXCR4	Refractory acute lymphoblastic leukemia	Phase 1	Plerixaflor	NCT01319864 (273)
RANKL	Breast cancer	Early phase 1	Denosumab	NCT02900469
RANKL	Bone metastases	Phase 1	JMT103	NCT03550508
TNF1α	Advanced cancer		Infliximab	(274)^a^
IL-17	Patients with relapsed and/or refractory multiple myeloma	Phase 1/1b	CJM112/anti-IL-17A antibody alone or in combination with drug: PDR001, anti-PD1 antibody	NCT03111992 (recruiting)
CCL2	Solid tumors	Phase 1	Carlumab	NCT01204996 (275)
CCL2	Prostate cancer	Phase 2	Carlumab	NCT00992186 (276)
VEGF	Many cancer types	Phase 1/2/3	Bevacizumab and others anti-VEGF drugs	(277, 278)

*^a^Due to the very high number of clinical trials targeting these cytokines, here we referred to review papers that provide an overview of the different experimentations*.

Here, we have provided a full overview about the pleiotropic role of Notch signaling dysregulation in tuning the expression and activity of a plethora of cytokines involved in the pathological interaction between tumor and TME.

This evidence together with the key role played by Notch dysregulation in several malignancies, suggests that a Notch-targeted approach may be sufficient to restore the physiological cytokine milieu, overcoming tumor-driven immune reprogramming, and improving patient’s overall survival.

Unfortunately, the majority of Notch-directed approaches are based on GSIs, whose “pan Notch”-blocking activity results in patient’s gastrointestinal toxicity due to intestine metaplasia (279, 280). Thus, a better dissection of the specific contribution of the different Notch ligands and receptors to the dysregulation of the cytokine milieu would help to better refine therapeutic strategies directed to restore the antitumor immune response, avoiding GSI-related side effects. At this purpose, recently a new generation of drugs has been developed such as monoclonal antibodies targeting Notch1 (281), Notch2/3 (282), Dll4 (283), or small molecules targeting Jagged1/2 (284, 285). These novel therapeutic strategies promise to specifically inhibit the dysregulated members of the Notch pathway, contributing to restore the normal activity of the immune system, and finally hampering tumor progression.

## Author Contributions

All the listed authors have provided a substantial, direct, and intellectual contribution to the work and approved it for the publication.

## Conflict of Interest Statement

MC-I is the CEO and Founder of Kiromic. LM is Vice President of R&D at Kiromic. All the other authors declare no competing interests.
